# Effects of time-restricted eating on metabolic panel and sleep profile in patients with type 2 diabetes: a meta-analysis

**DOI:** 10.3389/fnut.2026.1906991

**Published:** 2026-09-14

**Authors:** He Li, Zijiao Xue, Gao Wong, Lingli Hou

**Affiliations:** 1 Department of Neurosurgery, Taihe Hospital, Hubei University of Medicine, Shiyan, Hubei, China; 2 Department of Rehabilitation Medicine, Affiliated Hospital of North China University of Science and Technology, Tangshan, Hebei, China; 3 Department of Clinical Laboratory, Taihe Hospital, Hubei University of Medicine, Shiyan, Hubei, China

**Keywords:** meta-analysis, metabolic panel, systematic review, time-restricted eating, type 2 diabetes

## Abstract

**Background:**

Time-restricted eating (TRE), a novel dietary approach for the management of type 2 diabetes mellitus (T2DM), has been shown to affect the metabolic panel. Accordingly, this study aimed to evaluate the efficacy of the TRE on glycemic control, lipid profiles, anthropometric measurements, metabolic markers, and sleep-related outcomes in patients with T2DM.

**Methods:**

A systematic search was conducted in PubMed, Embase, Web of Science, and the Cochrane Library from inception up to May 2026. A random-effects model was used, and effect sizes are reported with 95% confidence intervals (CIs). Because of substantial heterogeneity, pooled estimates are presented as hypothesis-generating rather than confirmatory.

**Results:**

Thirteen studies (*n* = 544 participants) were included from 915 records. The pooled analysis suggested that TRE may be associated with a reduction in glycemic control (HbA1c; WMD: −0.46%; 95% CI: −0.91, −0.00; *p* = 0.048) and in central adiposity (WC; SMD: –0.55; 95% CI: −1.09, −0.02; *p* = 0.042); however, these findings are based on low- to moderate-certainty evidence with very high heterogeneity (*I*^2^ > 90% for most outcomes). No significant effects were observed for fasting blood glucose (FBG), homeostatic model assessment for insulin resistance (HOMA-IR), lipid profile, blood pressure, dietary intakes, or sleep outcomes (*p* > 0.05). The reduction in body mass index (BMI) was not robust; it was entirely driven by a single study, as omitting this trial in the leave-one-out sensitivity analysis rendered the pooled estimate non-significant. Subgroup analyses based on duration, sample size, and age were underpowered and did not explain the heterogeneity. Quality assessment showed that most RCTs had a low risk of bias, but the GRADE certainty of evidence was downgraded to low or moderate for all outcomes, mainly due to serious inconsistency and imprecision.

**Conclusion:**

TRE may offer a straightforward dietary approach that could have some benefits for glycemic control (HbA1c) and central adiposity (WC) in individuals with T2DM. However, due to substantial statistical heterogeneity, small sample sizes, the fragility of pooled estimates, and low-to-moderate GRADE certainty, the current evidence does not support a definitive clinical recommendation. For all other outcomes—weight, lipids, blood pressure, sleep, dietary composition, no consistent or robust evidence of an effect was found. High-quality, long-term RCTs are needed before firm conclusions can be drawn.

## 1 Introduction

Type 2 diabetes mellitus (T2DM) is a metabolic disorder characterized by hyperglycemia, insulin resistance, and impaired *β*-cell function (1, 2). Evidence indicates an increased prevalence of T2DM diagnoses as of 2024, with the total number of affected individuals estimated to reach 53 million by 2050 (3, 4). Moreover, the rising prevalence of T2DM and its associated increase in mortality underscore the urgent need for effective interventions to improve it. Although there has been significant progress in pharmacological approaches, lifestyle modifications, particularly dietary interventions, may also exert beneficial effects Dietary interventions have been shown to play a critical role in the management of T2DM (5). Similarly, exploring more effective and flexible dietary approaches remains unclear.

In this regard, intermittent fasting (IF) emerged its potential to improve metabolic panel in T2DM (6, 30). There are various types of IF, including alternate day fasting (ADF), twice weekly fasting (TWF), cyclical fasting, and time-restricted eating (TRE) (7, 8). Of these, TRE limits dietary intake to a specific daily eating window, ranging from 6 to 10 h, followed by fasting during the remaining hours, with non-caloric beverages permitted (9, 10). TRE has been shown to be easier to for individuals to adhere to. Previous studies have illustrated that TRE is associated with improved glycemic state, insulin sensitivity, anthropometric measurements, and metabolic risk factors (11–13). Moreover, previous studies have suggested that TRE may affect the metabolic features associated with T2DM, such as lipid profile, systemic inflammation, and sleep-related markers (14).

Therefore, the current systematic review and meta-analysis aimed to comprehensively evaluate the effects of TRE on glycemic control, obesity indices, lipid profiles, metabolic indices, dietary intakes, and sleep-related markers, which could provide a more realistic overview and judgment of TRE efficacy.

## 2 Materials and methods

This study was conducted in accordance with the Preferred Reporting Items for Systematic Reviews and Meta-Analysis (PRISMA) guidelines (15). Furthermore, its protocol has been registered on the PROSPERO International Register of Systematic Reviews (CRD42024515403).

### 2.1 Search strategy

A comprehensive search of PubMed, Scopus, Web of Science, Embase, and the Cochrane Library was performed from inception up to May 2026, without any language limitation. The search strategy for the predefined databases is provided in the Supplementary Table 1.

Search key terms included “Diabetes Mellitus,” “Type 2 Diabetes,” “T2DM,” “time restricted feeding,” “time restricted eating,” “TRE,” and “TRF.” In addition to database searching, we conducted citation tracking and hand searches of the reference lists of all included studies to identify additional eligible studies. To ensure a thorough retrieval of the literature and to minimize publication bias, we also searched grey literature sources, including conference papers and theses, in October. Preprints and unpublished trials were excluded according to our eligibility criteria. Furthermore, as our review focused on published data, records from trial registries were excluded. The search strategy is provided in Supplementary Table 1.

### 2.2 Inclusion and exclusion criteria

The inclusion criteria were as follows: Population: individuals clinically diagnosed with T2DM based on American Diabetes Association (ADA) established clinical criteria. Studies were included if T2DM diagnosis was based on established clinical criteria or physician diagnosis. Studies with mixed populations were included if the T2DM-specific data were presented; (1) intervention: TRE was used in the studies; (2) control: a normal diet (ND) or other non-TRE diet; (3) outcome: multiple measures including glycemic control, lipid profiles, anthropometric measurements, metabolic factors, sleep-related outcomes, and dietary intakes were assessed; and (4) study design: interventional studies, including both randomized controlled trials (RCTs) and non-randomized trials.

The exclusion criteria were as follows: (1) clinical trial protocols, conference abstracts, review studies, and animal studies, non-peer-reviewed publications were excluded; (2) studies with interventions other than TRE; (3) duplicate publications; and (4) publications written in languages other than English because full-text translation was not feasible.

### 2.3 Study selection and data extraction

All retrieved studies were imported into EndNote (version 21). The screening process was performed independently by two researchers. In the primary screening stage, studies were assessed based on their titles and abstracts. Subsequently, in the secondary screening stage, full texts of the remaining studies were evaluated for final inclusion. Any discrepancies were resolved by the authors with a third investigator. The extracted items included the first author’s name, publication year, location, study design, sample size, age, health condition, intervention type, and the duration of the intervention. Continuous outcomes were extracted as means and standard deviations (SDs) at baseline and at study endpoint. When only change-from-baseline values were reported, these values were extracted directly. For studies that did not report SDs, they were imputed using calculations from standard errors or confidence intervals. For change-from-baseline SDs that were not reported, we assumed a correlation coefficient of 0.5 between baseline and endpoint measurements, as recommended by the Cochrane Handbook, because this was considered to provide a conservative estimate of variance.

### 2.4 Quality assessment

The methodological evaluation was completed independently by two researchers. Any disagreements between the two reviewers were resolved through discussion and, when necessary, were consulted with a third investigator. RCTs were evaluated using the Cochrane risk of bias tool (ROB-2), which consists of the following domains: randomization procedures, intervention allocation, incomplete outcome data, measurement of outcomes, and selective outcome reporting. Each domain was scored as “low risk of bias,” “unclear,” and “high risk of bias.” For the single randomized crossover trial (20), we used the ROB-2 crossover tool, which includes additional signaling questions for period and carryover effects. The washout period (4 weeks) was judged as adequate, but carryover could not be completely ruled out. In addition, non-randomized controlled trials were evaluated using the ROBINS-I tool. This tool includes evaluating the study design, conduct, and reporting.

### 2.5 Statistical analysis

This study included RCTs, one crossover trial, and before–after studies (BAS). Accordingly, it is methodologically inappropriate to pool RCTs/crossover trials with BAS in a single meta-analytic estimate. All pooled analyses were conducted separately by study design: (1) parallel-group RCTs and crossover RCTs and (2) BAS. Meta-analyses were performed in STATA using random-effects models (*I*^2^ > 50% considered substantial heterogeneity) to account for anticipated between-study heterogeneity. For RCTs, continuous outcomes such as FBG, HOMA-IR, HbA1c, total cholesterol (TC), LDL-C, high density lipoprotein cholesterol (HDL C), aspartate aminotransferase (AST), alanine aminotransferase (ALT), systoli c blood pressure (SBP), and diastolic blood pressure (DBP), weight, BMI, WC, sleep duration, sleep latency, time in range (TIR), carbohydrate, protein, fat, fiber, sugar, and alcohol were pooled using weighted mean differences (WMD) for HbA1c and HOMA-IR (because they share the same units across studies) and standardized mean differences (SMD) for all other outcomes because different measurement scales were used. Heterogeneity was tested by Cochran’s *Q* test. An *I*^2^ value of >50% was considered indicative of substantial heterogeneity. Sensitivity analyses (leave-one-out method) and subgroup analyses explored heterogeneity sources. Publication bias was assessed where it was feasible.

## 3 Results

### 3.1 Screening and selection process

The present comprehensive systematic search identified 912 publications, and 3 studies were added through manual searching. Accordingly, 496 duplicate records were removed. Consequently, 356 studies were excluded based on title and abstract screening. Similarly, 63 remaining studies were evaluated using their full texts, and 51 studies were excluded for the following reasons: studies with a different population, studies with another type of fasting, and studies with a different design. Finally, 13 studies were included in the meta-analysis (Figure 1).

**Figure 1:**
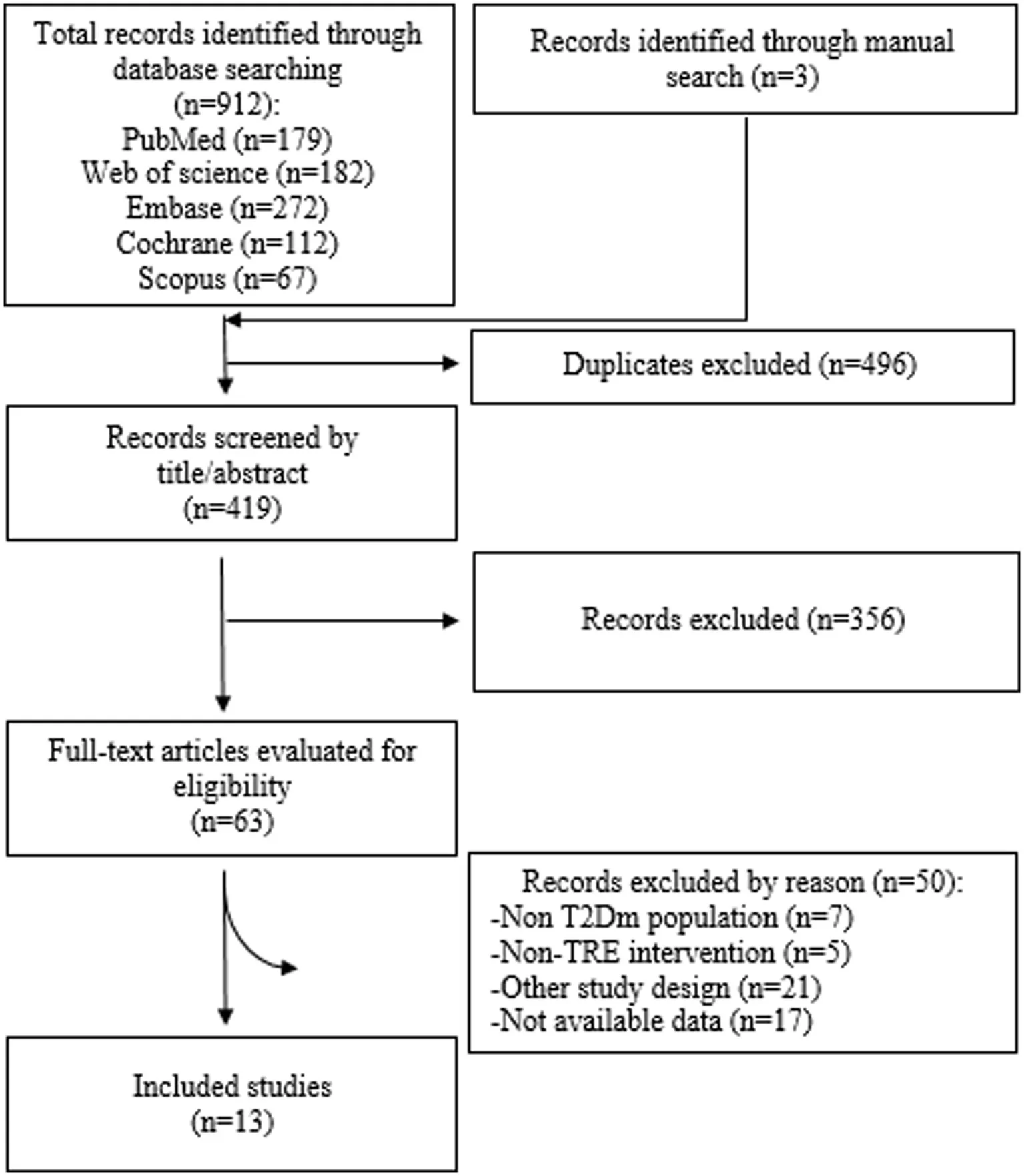
Flow diagram of study selection process.

### 3.2 Study characteristics

A total of 13 studies met the inclusion criteria. The basic characteristics of included studies are provided in Table 1. The publications ranged from 2017 to 2025. Our target population was patients with T2DM. This study included seven RCTs, one randomized crossover trial, and five before-after studies (BASs). All included studies used TRE as the intervention, with durations ranging from 4 to 18 weeks.

**Table 1:** Characteristics of included studies.

Author/year	Country	Type of study	Sample size	Gender	Age(year)	Intervention	Health status	Duration
Parr et al. (29)	Australia	RCT	TRE:22; C:21	Both	55.6 ± 8.4	9-h eating window (1000-1900 h)	T2DM	6 months(2-week baseline)
Che et al. (17)	China	RCT	TRE:54; C:50	Both	TRE:48.21 ± 9.32; C:48.78 ± 9.56	10-h eating window (0800-1800 h)	T2DM	14 weeks (2-week baseline, 12-week intervention)
Pavlou et al. (19)	USA	RCT	TRE:25; C: 25	Both	TRE:56 ± 13; C:54 ± 11	8-h eating window (1200-2000 h)	T2DM	6 months
Pavlou et al. (16, 18)	USA	RCT	TRE:25; C: 25	Both	TRE:56 ± 13; C:54 ± 11	8-h eating window(1200-2000 h)	T2DM	6 months
Suthutvoravut et al. (11)	Thailand	RCT	TRE:26; C: 24	Both	54.6 ± 8.1	9-h window (between 8:00 a.m. and 5:00 p.m.)	T2DM	12 weeks
Quist et al. (35)	Denmark	RCT	TRE:50; C: 50	Both	59	eating window (eating and drinking [except for water]) of 12 h per day or more every day and of 14 h per day or more at least 1 day per week	T2DM	12 weeks
Tricò et al. (37)	Italy	RCT	TRE:12; C: 11	Both	67.2 ± 7.9	–	T2DM	12 weeks
Kramer et al. (20)	Canada	RCT (cross-over)	39	Both	56.3 ± 9.4	4-h eating window (1600- 2000 h or 1700-2100 h)	T2DM	TRE(8w) → washout period(4w) → ND(8w)
Parr (31)	Australia	BAS	19	Both	50.2 ± 8.9	9-h eating window(1000-1900 h)	T2DM	4 weeks
Fu et al. (36)	China	BAS	18	Both	49.7 ± 9.00	10-h TRE	T2DM	12 weeks
Parr et al. (32)	Australia	BAS	19	Both	50.2 ± 8.9	9-h eating window(1000-1900 h)	T2DM	4 weeks
Zimmermann et al. (33)	USA	BAS	19	Both	64.7 ± 8.3	6–8-h eating window and a lower carbohydrate diet (≤30 g/day)	T2DM	6 months
Arnason et al. (34)	Canada	BAS	10	NR	53.8 ± 9.11	4-6 h eating window	T2DM	6 weeks (2-week baseline, 2-week intervention, and 2-week follow-up)

NR, not report; BAS, before–after study.

### 3.3 Quality assessment

The risk of bias for seven included RCTs was evaluated using the ROB-2 tool (Table 2). Accordingly, five studies were rated as having a lower risk of bias, and two studies were rated as unclear.

**Table 2:** Quality assessment of RCT studies.

Author/year	Bias arising from randomization process	Bias due to deviation from intended interventions	Bias due to missing outcomes data	Bias in the measurement of the outcome	Bias in selection of reported results	Bias due to period and carryover effect	Overall risk of bias
Parr et al. (29)	L	L	L	L	L	–	L
Che et al. (17)	L	L	L	L	L	–	L
Pavlou et al. (19)	U	U	L	L	L	–	U
Suthutvoravut et al. (11)	L	L	L	L	L	–	L
Quist et al. (35)	L	L	L	L	L	–	L
Tricò et al. (37)	L	L	L	L	L	–	L
Pavlou et al. (16, 18)	U	L	L	L	L	–	U

For the crossover trial (20), we used the appropriate ROB-2 crossover tool. Randomization, missing outcome data, measurement of the outcome, and selection of the reported result had a low risk of bias. However, period and carryover effects, as well as deviations from intended interventions, were rated as having a high risk of bias. Furthermore, quality assessment for BAS studies was conducted using the ROBINS-I tool. However, two studies were rated as having a lower risk of bias, one had a higher risk of bias, and one was unclear (Table 3).

**Table 3:** Quality assessment of before–after studies.

Author/year	Bias due to confounding	Bias due to selection of participants	Bias in classification of intervention	Bias due to deviation from intended interventions	Bias due to missing data	Bias in measurement of outcome	Bias in selection of reported result	Overall risk of bias
Parr et al. (32)	L	L	L	L	L	L	L	L
Fu et al. (36)	L	L	L	L	L	L	L	L
Arnason et al. (34)	U	U	L	L	L	L	L	U
Zimmermann et al. (33)	H	L	L	L	L	L	L	H

### 3.4 Glycemic parameters

#### 3.4.1 FBG

The present meta-analysis included five studies with 261 T2DM patients for FBG values (Table 4). Accordingly, it has been demonstrated that TRE was not successful in affecting the FBG level significantly (SMD: –0.63, 95% CI: −1.88, 0.63, *p* = 0.328; τ^2^ = 1.95, *I*^2^ = 95.1%, *p* < 0.001) (Figure 2A). Given the high heterogeneity, this result should be interpreted with caution. However, subgroup analysis based on the duration showed that neither the short-term not the long-term studies were effective in reducing FBG levels (Table 5). In addition, sensitivity analysis revealed that excluding any single study had no effect on the pooled effect estimate for FBG (Supplementary Figure 1A). Moreover, Egger’s test showed no evidence of publication bias (*p* = 0.623); however, the small number of included studies for the FBG outcome warrants cautious interpretation (Supplementary Figure 1B).

**Table 4:** Summary of RCT findings and quality of evidence assessment using the GRADE approach.

Outcomes	No of patients (meta-analysis)	EF (95% CI)	Risk of bias^1^	Inconsistency^2^	Indirectness^3^	Imprecision^4^	Publication bias^5^	Quality of evidence^6^
FBG	261 (5)	-0.63 (−1.88, 0.63)	Not serious	Serious	Not serious	Serious	Not serious	Moderate
HbA1c	443 (7)	−1.10 (−2.16, −0.03)	Not serious	Serious	Not serious	Not Serious	Not serious	Downgraded
HOMA-IR	209 (4)	−0.23 (−0.80, 0.33)	Not serious	Not serious	Not serious	Serious	Not serious	Downgraded
TC	193 (3)	−0.74 (−2.64, 1.16)	Not serious	Serious	Not serious	Serious	Not serious	Moderate
LDL-C	282 (4)	−0.17 (−1.22, 0.87)	Not serious	Serious	Not serious	Serious	Not serious	Moderate
HDL-C	154 (2)	0.03 (−0.53, 0.58)	Not serious	Serious	Not serious	Serious	Not serious	Moderate
TG	193 (3)	−0.70 (−1.48, 0.08)	Not serious	Serious	Not serious	Serious	Not serious	Moderate
Weight	400 (6)	0.74 (−1.43, 2.92)	Not serious	Serious	Not serious	Serious	Not serious	Moderate
BMI	259 (5)	−1.35 (−2.68, −0.01)	Not serious	Serious	Not serious	Not serious	Not serious	Downgraded
WC	162 (3)	−0.55 (−1.09, −0.02)	Not serious	Not serious	Not serious	Not serious	Not serious	High
SBP	89 (2)	−0.24 (−0.66, 0.18)	Not serious	Not serious	Not serious	Serious	Not serious	Downgraded
DBP	89 (2)	−0.21 (−0.63, 0.21)	Not serious	Not serious	Not serious	Serious	Not serious	Downgraded
Cal	286 (4)	−1.41 (−3.47, 0.65)	Not serious	Serious	Not serious	Serious	Not serious	Moderate
CHO	297 (4)	−1.14 (−2.53, 0.25)	Not serious	Serious	Not serious	Serious	Not serious	Moderate
Pro	297 (4)	0.88 (−0.29, 2.06)	Not serious	Serious	Not serious	Serious	Not serious	Moderate
Fat	297 (4)	0.62 (−0.09, 1.33)	Not serious	Serious	Not serious	Serious	Not serious	Moderate
Fiber	154 (2)	0.00 (−0.32, 0.32)	Not serious	Not serious	Not serious	Serious	Not serious	Downgraded
Sugar	197 (3)	−1.50 (−3.82, 0.82)	Not serious	Serious	Not serious	Serious	Not serious	Moderate
Alcohol	93 (2)	0.00 (−0.41, 0.41)	Not serious	Not serious	Not serious	Serious	Not serious	Downgraded

^1^ Risk of bias based on the Cochrane risk of bias tool. ^2^ Downgraded if there was a substantial unexplained heterogeneity (*I^2^* > 50%, *p* < 0.10) that was not explained by subgroup analyses. ^3^ Downgraded if there were factors present relating to the participants, interventions, or outcomes, which restrict the overall generalizability of the results. ^4^ Downgraded if the 95% confidence interval (95% CI) crossed the minimally important difference (MID). ^5^ Downgraded if there was evidence of publication bias using a funnel plot. ^6^ Since all included studies were randomized controlled trials, the certainty of the evidence was graded as high for all outcomes by default and then downgraded based on pre-specified criteria.

**Figure 2:**
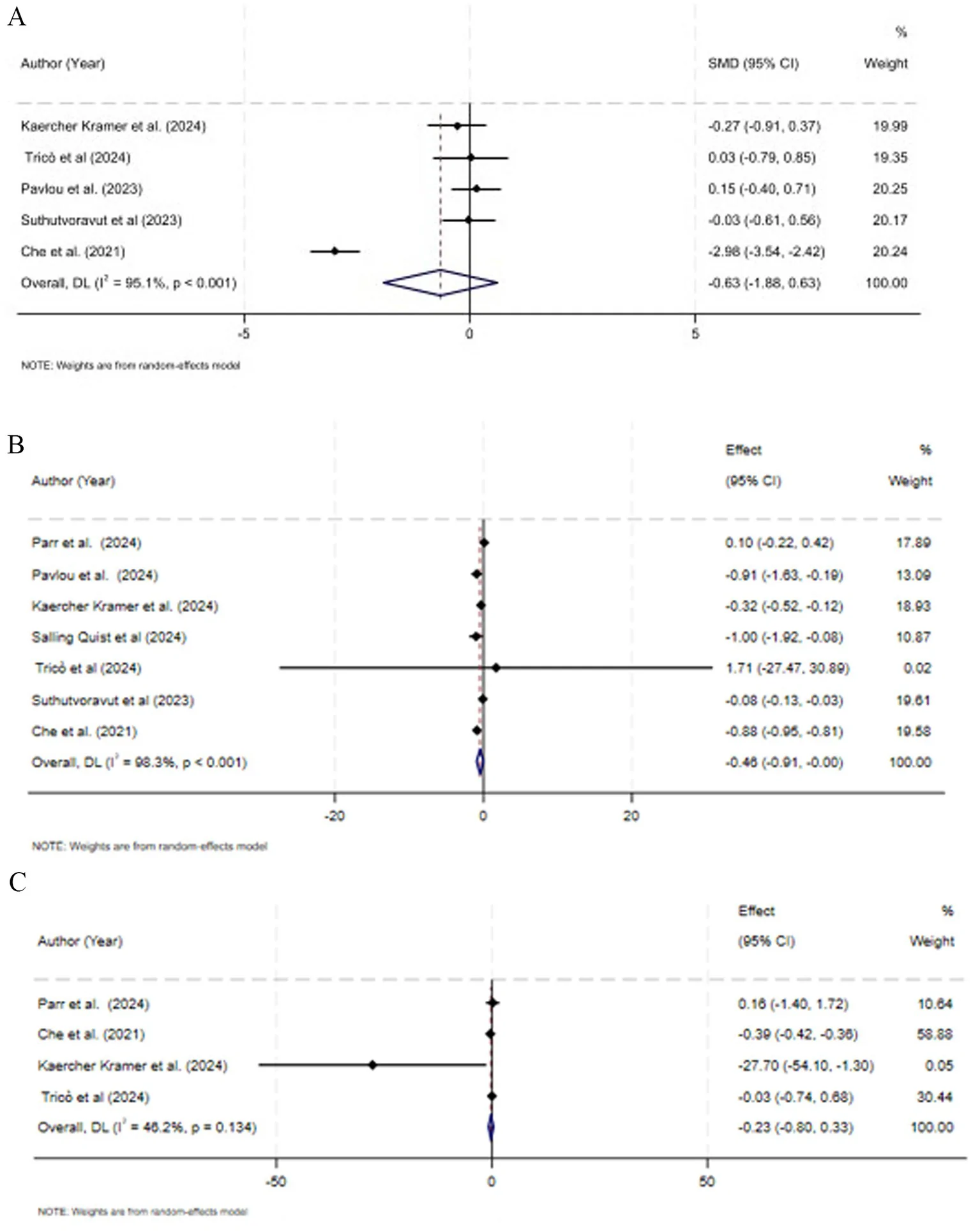
**(A)** Forest plot of TRE intervention on the FBG level displays study-specific mean differences with 95% CI using a random-effects model. **(B)** Forest plot of TRE intervention on the HbA1c level displays study-specific mean differences with 95% CI using a random-effects model. **(C)** Forest plot of TRE intervention on the HOMA-IR level displays study-specific mean differences with 95% CI using a random-effects model.

**Table 5:** Pooled estimate effects of TRE on the various outcomes across different subgroups.

Group	No. of comparisons	ES (95% CI)	*p*-value	Tau2	*I*^2^ (%)	P-heterogeneity
FBG						
Duration						
<12 weeks	1	−0.27 (−0.91, 0.37)	0.408	–	0.0%	<0.001
≥12 weeks	4	−0.71 (−2.30, 0.87)	0.379	2.51	96.2%	<0.001
Sample size						
<50	4	−0.02 (−0.33, 0.29)	0.901		0.0	0.809
>50	1	−2.98 (−3.54, −2.42)	<0.001		0.0	<0.001
Age						
<50	4	−0.02 (−0.33, 0.29)	0.901	2.51	0.0	0.809
>50	1	−2.98 (−3.54, −2.42)	<0.001	–	0.0	<0.001
HbA1c						
Duration						
<12 weeks	6	−1.17 (−2.45, 0.11)	0.074	2.30	96.0%	<0.001
≥12 weeks	1	−0.70 (−1.27, −0.13)	0.017	-	0.0%	<0.001
Sample size						
<50	4	−0.42 (−1.03, 0.19)	0.181		69.9	0.019
>50	3	−2.03 (−4.42, 0.37)	0.098		98.1	<0.001
Age						
<50	1	−4.99 (−5.78, −4.21)	<0.001		0.0	<0.001
>50	6	−0.48 (−0.83, −0.12)	0.009		53.0	0.059
HOMA-IR						
Duration						
<12 weeks	1	−0.68 (−1.34, −0.02)	0.043	–	0.0%	<0.001
≥12 weeks	3	−1.81 (−5.20, 1.58)	0.295	8.81	98.4%	<0.001
Sample size						
<50	3	−0.22 (−0.70, 0.25)	0.359		31.5	0.232
>50	1	−5.49 (−6.33, −4.64)	<0.001		0.0	<0.001
Age						
<50	1	−5.49 (−6.33, −4.64)	<0.001		31.5	0.232
>50	3	−0.22 (−0.70, 0.25)	0.359		0.0	<0.001
Weight						
Duration						
<12 weeks	1	−1.69 (−2.44, −0.95)	<0.001	–	98.3%	<0.001
≥12 weeks	5	1.57 (−1.11, 4.26)	0.251	8.50	0.0%	<0.001
Sample size						
<50	3	−1.01 (−1.95, −0.08)	0.033		79.4	0.008
>50	3	4.95 (−1.09, 10.98)	0.108		99.1	<0.001
Age						
<50	1	−5.64 (−6.51, −4.78)	<0.001		0.0	<0.001
>50	5	1.50 (−0.37, 3.38)	0.117		96.6	<0.001
BMI						
Duration						
<12 weeks	1	−1.67 (−2.41, −0.93)	<0.001	–	0.0%	<0.001
≥12 weeks	4	−1.67 (−2.41, −0.93)	0.137	2.80	96.2%	<0.001
Sample size						
<50	4	−0.72 (−1.35, −0.10)	0.023		70.7	0.017
>50	1	−3.81 (−4.46, −3.16)	<0.001		0.0	<0.001
Age						
<50	1	−3.81 (−4.46, −3.16)	<0.001		0.0	<0.001
>50	4	−0.72 (−1.35, −0.10)	0.023		70.7	0.017
WC						
Duration						
<12 weeks	1	−0.77 (−1.43, −0.11)	0.023	–	0.0%	<0.001
≥12 weeks	2	−0.40 (−1.31, 0.51)	0.388	0.30	69.8%	0.069

#### 3.4.2 HbA1c

Seven RCTs (*n* = 443) showed a statistically significant reduction (WMD: −0.46%; 95% CI: −0.91 to −0.00, *p* = 0.048; *I*^2^ = 98.3%, *p* < 0.001) (Figure 2B). However, given the very high heterogeneity and the fact that the result was not consistently robust across sensitivity analyses, this finding should be interpreted with extreme caution and considered hypothesis-generating rather than confirmatory. Furthermore, subgroup analysis showed that long-term intervention was not able to affect the HbA1c level (*p* > 0.05) (Table 5). Sensitivity analysis indicated that no single study had a significant effect on HbA1c (Supplementary Figure 1C). No significant publication bias was detected in the analysis (*p* = 0.623) (Supplementary Figure 1D).

#### 3.4.3 HOMA-IR

Four studies (209 individuals) have assessed the efficacy of TRE on the HOMA-IR level, and the pooled analysis indicated no significant effect on the HOMA-IR level (WMD: -0.23, 95% CI: −0.80, 0.33, *p* = 0.415; τ^2^ = 0.14, *I*^2^ = 46.2%, *p* = 0.134) (Figure 2C). Moreover, long-term TRE intervention had no effect on HOMA-IR levels, whereas short-term intervention in one study was effective (Table 5). The leave-one-out approach found no study that could alter the overall results (Supplementary Figure 1E). Egger’s test indicated no significant effect for HOMA-IR value (*p* = 0.350) (Supplementary Figure 1F).

#### 3.4.4 TIR and medication effect score (MES)

These outcomes were reported in limited studies to permit the meta-analysis. Continuous glucose monitoring (CGM) further confirmed that TRE had no effect on the time in range (TIR) in one included study (16). Additionally, the effect of TRE on the dose or number of diabetes medications presented as MES was evaluated in the two studies, and Che et al. (17) reported reduced MES following TRE, while Pavlou et al. showed no significant effect of TRE (16).

### 3.5 Anthropometric parameters

#### 3.5.1 Weight

The combined effect of six studies involving 400 T2DM patients showed no significant effect on weight (SMD: 0.74, 95% CI: −1.43, 2.92, *p* = 0.501; τ^2^ = 6.65, *I*^2^ = 97.9%, *p* < 0.001) (Figure 3A). Given the very high heterogeneity, this result should be interpreted with caution. Subgroup analysis based on the intervention duration of was conducted and revealed that TRE lasting ≥12 weeks had no significant effect, while one study with a lower duration was associated with a favorable effect (Table 5). Sensitivity analysis showed that the exclusion of any single study did not sufficiently alter the weight results (Supplementary Figure 2A). Although Egger’s test showed no publication bias (*p* = 0.649), this finding should be interpreted with caution (Supplementary Figure 2B).

**Figure 3:**
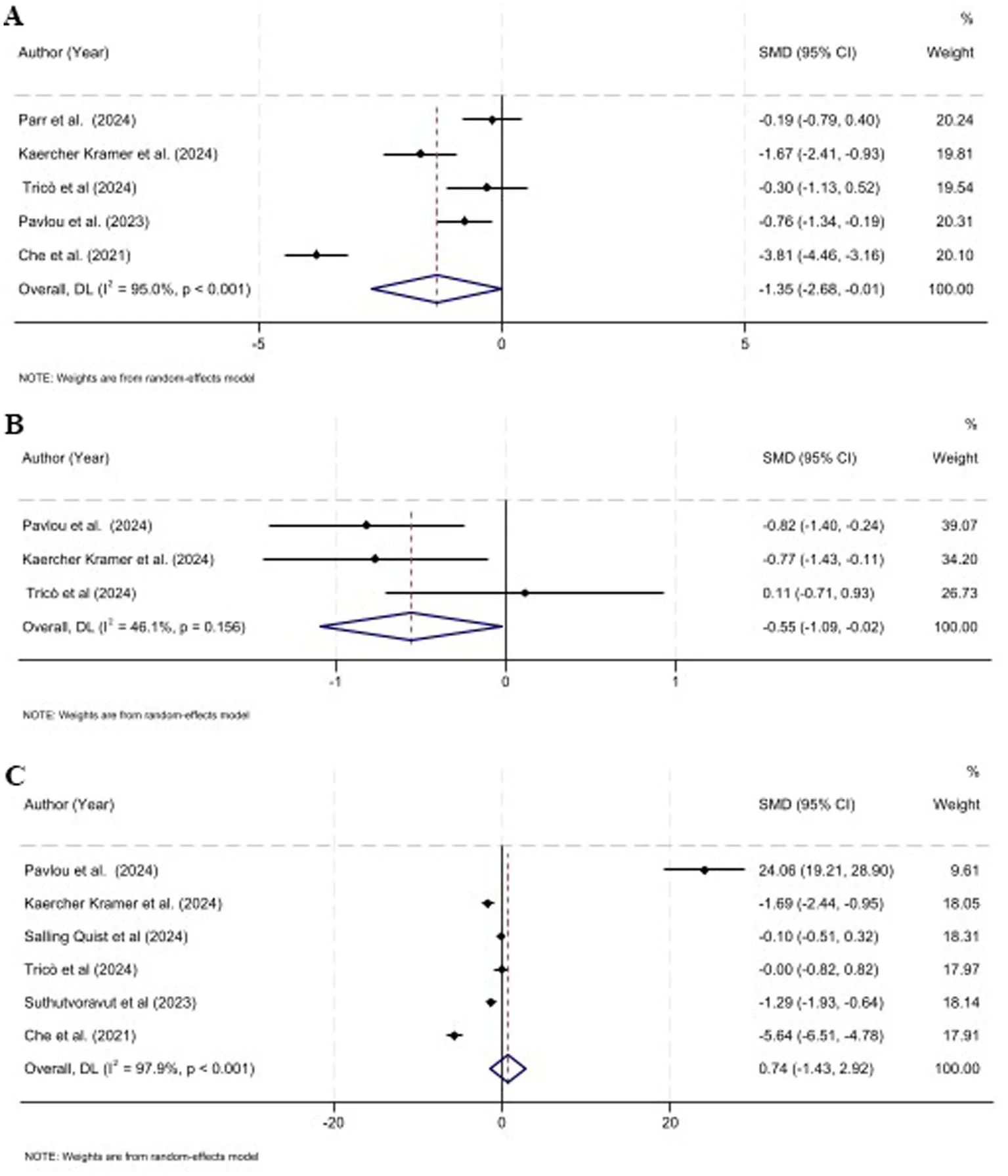
**(A)** Forest plot of TRE intervention on the weight level displays study-specific mean differences with 95% CI using a random-effects model. **(B)** Forest plot of TRE intervention on the BMI level displays study-specific mean differences with 95% CI using a random-effects model. **(C)** Forest plot of TRE intervention on the WC level displays study-specific mean differences with 95% CI using a random-effects model.

#### 3.5.2 BMI

Five studies involving 259 participants demonstrated that TRE significantly reduced BMI in T2DM patients (SMD: –1.35, 95% CI: −2.68, −0.01, *p* = 0.048; τ^2^ = 2.19, *I*^2^ = 95.0%, *p* < 0.001) (Figure 3B). However, the reduction in BMI was fragile and driven entirely by a single study (18); after excluding this trial in the leave-one-out sensitivity analysis, the pooled effect remained significant (SMD: -0.72, 95% CI: −1.35 to −0.10, *p* = 0.023) (Supplementary Figure 2C). Given this fragility and the very high heterogeneity (I^2^ = 95.0%), there is no robust evidence that TRE reduces BMI in patients with T2DM. Similarly, the subgroup analysis showed that a ≥ 12-week TRE intervention had no effect on BMI (Table 5). Whereas exclusion of Pavlou et al.’s study changed the effect of TRE on BMI to be non-significant (Supplementary Figure 2C). However, given the very high heterogeneity and the fragility of the pooled estimate, there is no robust evidence that TRE reduces BMI in T2DM. Furthermore, there was no sign of publication bias using Egger’s test (*p* = 0.926) (Supplementary Figure 2D).

#### 3.5.3 WC

The pooled effect of three RCTs (162 T2DM subjects) showed that TRE significantly decreased WC (SMD: –0.55, 95% CI: −1.09, −0.02, *p* = 0.042; τ^2^ = 0.10, *I*^2^ = 46.1%, *p* = 0.156) (Figure 3C). Although the heterogeneity was low, the pooled estimate was based on only three studies, making it insufficiently stable. Two studies with longer durations (≥12 weeks) showed no significant effect on WC (Table 5). Sensitivity analysis showed that the exclusion of two studies resulted in a different effect on the WC (19, 20) (Supplementary Figure 2E). Egger’s test showed no evidence of publication bias (*p* = 0.224).

### 3.6 Blood lipids

#### 3.6.1 TC

The combined effect of 3 RCTs involving 193 participants indicated that TRE has no significant effect on the TC level (SMD: -0.74, 95% CI: −2.64, 1.16, *p* = 0.446, *I*^2^ = 97.0%, *p* < 0.001) (Figure 4); this result should be interpreted with caution.

**Figure 4:**
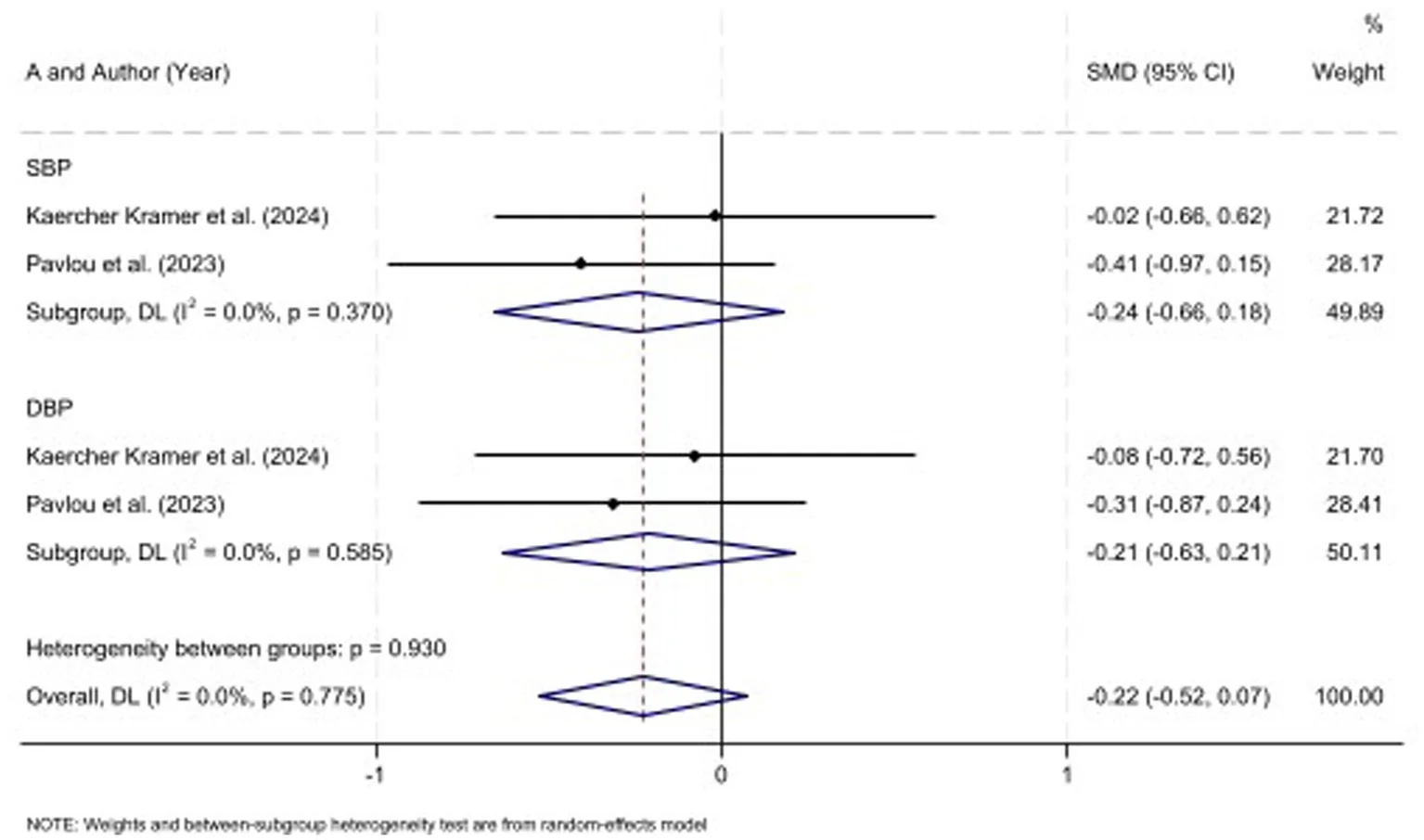
Forest plot of TRE intervention on the TC, LDL-C, HDL-C, and TG levels displays study-specific mean differences with 95% CI using a random-effects model.

#### 3.6.2 LDL-C

The combined effect of four RCTs involving 282 subjects indicated that TRE has no significant effect on the LDL-C level (SMD: -0.17, 95% CI: −1.22, 0.87, *p* = 0.745, *I*^2^ = 94.2%, *p* < 0.001) (Figure 4); this result should be interpreted with caution.

#### 3.6.3 HDL-C

The overall effect of two studies (154 individuals) showed no significant effect of TRE on the LDL-C level (SMD: 0.03, 95% CI: −0.53, 0.58, *p* = 0.928, *I*^2^ = 63.0%, *p* = 0.999) (Figure 4).

#### 3.6.4 TG

The final effect of three studies encompassing 193 T2DM patients showed no significant effect of TRE intervention on the TG level (SMD: -0.70, 95% CI: −1.48, 0.08, *p* = 0.077, *I*^2^ = 84.1%, *p* = 0.002) (Figure 4); this result should be interpreted with caution.

### 3.7 Blood pressure and liver markers

#### 3.7.1 BP

The pooled effect of two studies revealed no significant effect on SBP (SMD: –0.24, 95% CI: −0.66, 0.18, *p* = 0.268, *I*^2^ = 0.0%, *p* = 0.370) and DBP (SMD: –0.21, 95% CI: −0.63, 0.21, *p* = 0.324, *I*^2^ = 0.0%, *p* = 0.585) (Figure 5).

**Figure 5:**
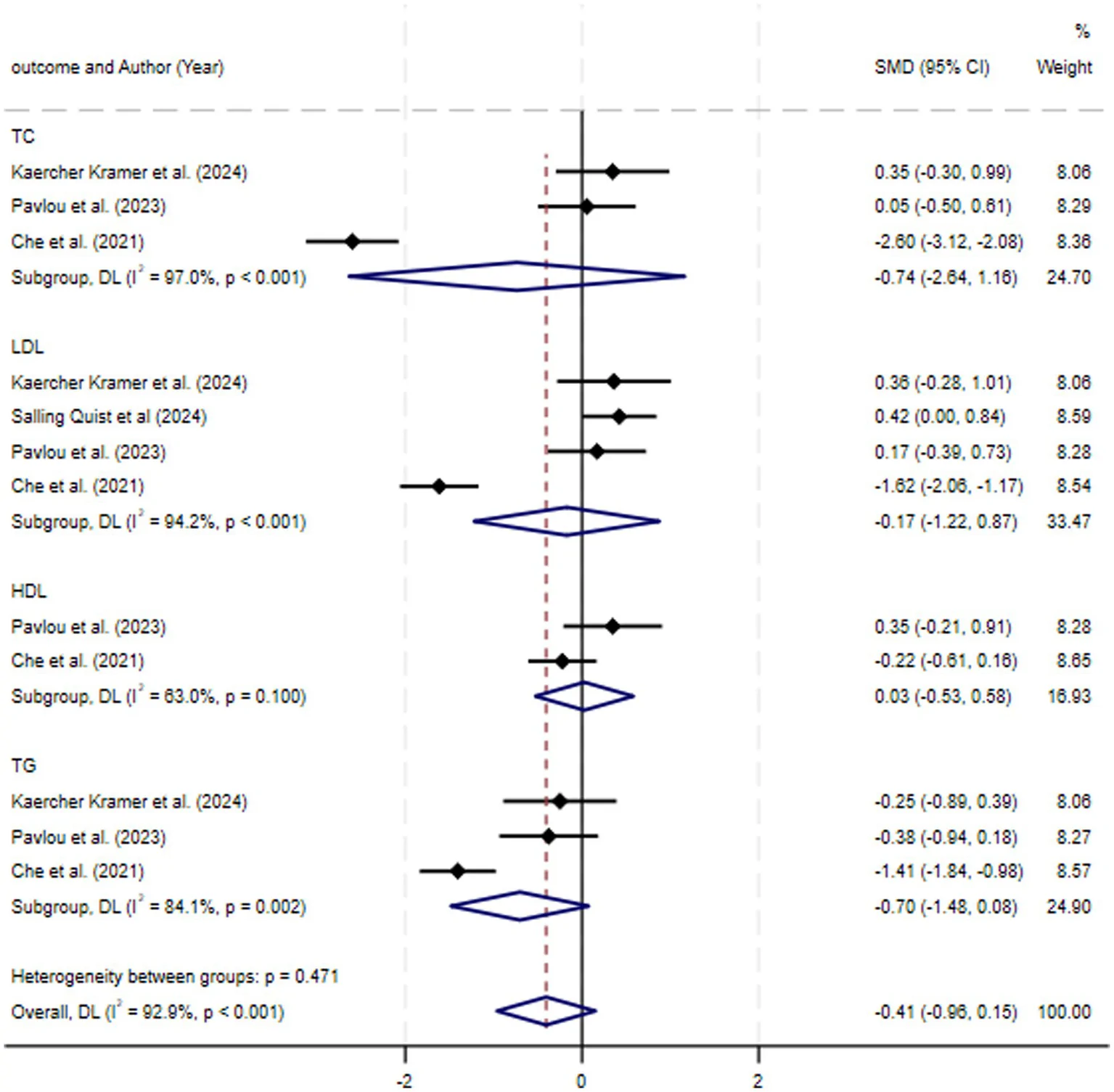
Forest plot of TRE intervention on the SBP and DBP level displays study-specific mean differences with 95% CI using a random-effects model.

#### 3.7.2 AST and ALT

These outcomes were reported in too few studies to permit a meta-analysis. One study evaluated AST and ALT and reported no significant change in AST but a significant reduction in ALT. This evidence is very limited and should be interpreted cautiously.

### 3.8 Dietary intakes

Dietary intake outcomes were reported in a limited number of RCTs with high statistical heterogeneity and wide confidence intervals. Therefore, these analyses are exploratory and underpowered. The pooled effect of TRE intervention in RCTs on calorie (SMD: –1.41, 95% CI: −3.47, 0.65, *p* = 0.180, *I*^2^ = 98.0%, *p* < 0.001), carbohydrate (SMD: –1.14, 95% CI: −2.53, 0.25, *p* = 0.108, *I*^2^ = 94.6%, *p* < 0.001), protein (SMD: 0.88, 95% CI: −0.29, 2.06, *p* = 0.140, *I*^2^ = 92.9%, *p* < 0.001), fat (SMD: 0.62, 95% CI: −0.09, 1.33, *p* = 0.086, *I*^2^ = 81.8%, *p* = 0.004), fiber (SMD: 0.0, 95% CI: −0.32, 0.32, *p* = 1.00, *I*^2^ = 0.0%, *p* = 1.00), sugar (SMD: –1.60, 95% CI: −3.82, 0.82, *p* = 0.206, *I*^2^ = 97.7%, *p* < 0.001), and alcohol (SMD: 0.00, 95% CI: −0.41, 0.41, *p* = 1.00, *I*^2^ = 0.0%, *p* = 1.00) intake was not significant (Figure 6). Based on the low number of included studies and high heterogeneity, there is no consistent evidence that TRE affects any dietary intake variable, though a clinically meaningful effect cannot be ruled out.

**Figure 6:**
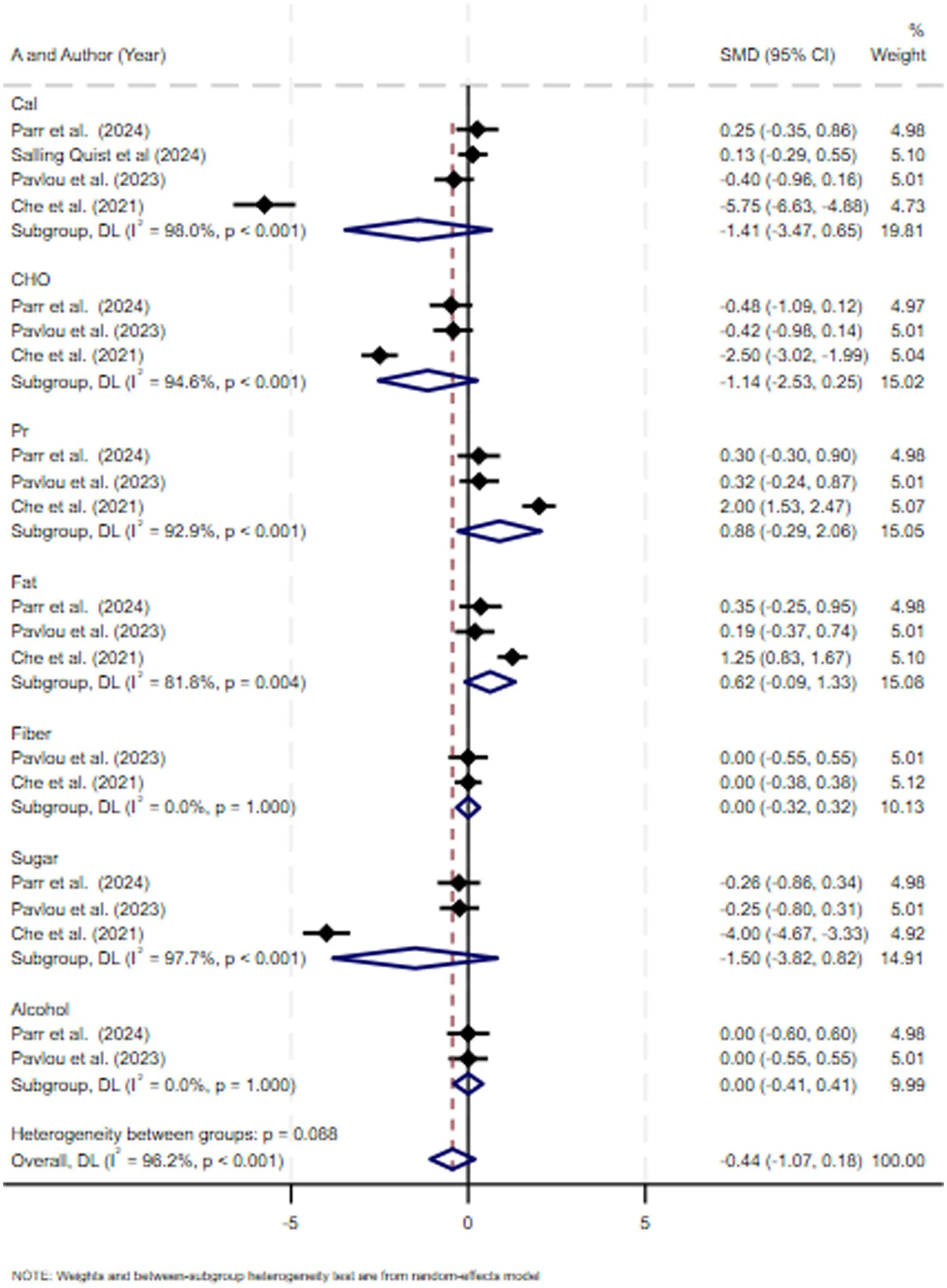
Forest plot of TRE intervention on the dietary intakes level displays study-specific mean differences with 95% CI using a random-effects model.

### 3.9 Sleep markers

Pavlou et al. assessed the effect of TRE on sleep-related outcomes such as sleep duration and sleep latency (19). No conclusion can be drawn about whether TRE affects sleep outcomes; therefore, studies with larger sample sizes are needed.

### 3.10 Results of BAS model studies

It has been shown that TRE could reduce the HbA1c level with no effect on the FBG, HOMA-IR (Supplementary Figure 3), weight, BMI, and WC (Supplementary Figure 4) level (*p* > 0.05) in the BAS model. Furthermore, Fu et al. have evaluated the effect of TRE on the lipid profile in a BAS model, and the effects on TC, LDL-C, HDL-C, and TG are presented in Supplementary Figure 5. Moreover, AST and ALT in the BAS model were not affected by TRE intervention (Supplementary Figure 6). Similarly, no significant effect of TRE on dietary intake was observed in the BAS model (Supplementary Figure 7), nor on sleep-related outcomes (Supplementary Figure 8).

## 4 Discussion

The present comprehensive systematic review and meta-analysis evaluated the efficacy of the TRE intervention for glycemic control, anthropometric indices, lipid profile, blood pressure and liver markers, sleep-related outcomes, and dietary intakes in patients with T2DM. Our findings suggest that TRE may significantly reduce HbA1c, with a pooled WMD of −0.46% (95% CI: −0.91 to −0.00), which meets the commonly accepted minimal clinically important difference (MCID) for HbA1c (typically 0.3–0.5%). However, the very high heterogeneity (I^2^ = 98.3%) indicates that the effect varies substantially across individuals and studies, and the GRADE certainty of evidence was moderate due to serious inconsistency. Therefore, these results should be considered hypothesis-generating rather than confirmatory. No significant effects were observed for FBG or HOMA-IR. It is worth noting that there was substantial heterogeneity and moderate certainty of evidence, suggesting that these findings should be interpreted with caution. Furthermore, all these pooled effect sizes are better considered as hypothesis-generating.

Our results partially align with recent reviews. Rovira-Llopis et al. reported no significant effect of TRE on FBG, consistent with our findings, although their meta-analysis included mixed populations (T2DM and non-diabetic) (21). Zeng et al. found 5:2 fasting to be superior to TRE for HbA1c and weight reduction. However, their network meta-analysis focused on various IF regimens rather than TRE specifically and did not assess sleep, dietary composition, or liver markers (22). Our review expands on this literature by providing the most complete metabolic panel to date, specifically for T2DM patients—including dietary intake, sleep performance, and ALT—while analyzing ex-ante studies separately to avoid combining them with RCTs and using the GRADE for formal assurance. This methodological approach allows us to be more cautious and conclude that the current evidence is not yet sufficient to support a definitive clinical recommendation. These effects may be mediated by calorie regulation (23) and optimization of circadian rhythms (24, 25). TIR was recognized as a key glycemic target, although its effect was uncertain due to limited data. From a clinical perspective, the observed reduction in HbA1c is consistent with thresholds considered relevant for the treatment of T2DM. However, the moderate certainty, high variability, and small sample size preclude a final conclusion.

Several statistically significant findings warrant caution. The BMI reduction was driven by a single study (18); excluding it rendered the pooled effect non-significant. The HbA1c estimate, though nominally significant, was undermined by substantial variability (*I*^2^ = 98.3%). Even the waist circumference reduction, despite lower heterogeneity, rested on only three studies. Thus, our results are exploratory and do not support definitive clinical conclusions. Taken together, these results highlight that our analysis is exploratory and cannot be interpreted as providing conclusive evidence of a clinically relevant benefit.

Moreover, a significant reduction was shown in BMI and waist circumference in patients with T2DM following TRE. This favorable finding is associated with insulin sensitivity, which contributes to delayed disease progression; it has been shown that TRE is not associated with weight changes (9). It seems that TRE may modify the circadian rhythms, endocrine pathways, and metabolic features through improved insulin sensitivity (26). Overall, this finding suggests that TRE may have metabolic benefits beyond simple calorie reduction. On the other hand, waist circumference is also associated with insulin sensitivity, inflammation, and cardiovascular risk. However, after removing Pavlou’s study (18), the effect was no longer significant on BMI. Given the high heterogeneity and the fragility of the pooled estimate, there is no robust evidence that TRE reduces BMI in T2DM.

However, this study demonstrated that TRE was not sufficiently potent to significantly affect lipid profile markers. The heterogeneity in the TRE protocol may account for this finding. Furthermore, lipid markers are more sensitive to the quality of dietary fats, which TRE does not affect. Evidence shows that TRE may have more pronounced effects on glycemic control rather than lipid profile, and lipid markers may be dependent on long-term intervention.

The observed reduction in ALT suggests that TRE may improve hepatic metabolic health. ALT is a liver injury marker and is elevated in individuals with T2DM. However, because this effect was reported in only one study, the evidence remains limited, and the finding should be interpreted with caution.

There are no significant changes in sleep duration or sleep latency in the included studies, and the BAS model showed no significant effects, which suggests that TRE may not have a potent effect on sleep-related outcomes in patients with T2DM. TRE may not sufficiently alter circadian rhythms to affect sleep, and heterogeneity about TRE intervention may be responsible for this controversy. Several studies have found that TRE reduces calorie intake with no changes in the macronutrient composition (27, 28).

The before-and-after studies, while analyzed separately, inherently carry a lower level of evidence due to their non-randomized design, lack of concurrent control groups, and higher susceptibility to confounding. Accordingly, our main conclusions are based on evidence from the RCT, and the BAS findings are used only as additional context, not as a basis for final decisions.

There are several limitations in this study that need to be addressed. First, the small number of included studies for each outcome (often 2–5) reduces the power and stability of pooled estimates. Second, we observed very high statistical heterogeneity (*I*^2^ > 90% for most outcomes). Subgroup analyses by duration, sample size, and age were underpowered and uninformative given the limited number of eligible trials. Heterogeneity likely reflects protocol diversity, varying eating windows (4–18 weeks), early versus late intake, caloric variability, medication adjustments, and disparate baseline phenotypes that precluded formal testing. Accordingly, our findings are hypothesis-generating rather than confirmatory and warrant validation in larger prospective cohorts. Third, the GRADE certainty of evidence was downgraded to low or moderate for all outcomes, primarily due to serious inconsistency and imprecision. Fourth, mixing different study designs (RCTs, one crossover with carryover risk, and before-after studies) adds more uncertainty—and although we analyzed BAS separately, their lower level of evidence indicates that these studies should receive less emphasis in the overall interpretation. Fifth, publication bias assessment was limited by the small number of studies; funnel plots were used only descriptively. Sixth, imputation of missing SDs using a correlation coefficient of 0.5 may have introduced some bias. Finally, sleep outcomes were self-reported in most studies.

### 4.1 Clinical and research implications

Clinicians should not currently prescribe TRE as a reliable strategy for improving metabolic performance beyond the potential glucose-lowering effects, which are themselves uncertain. Future research should provide priority to large, well-designed RCTs with standardized TRE protocols (early versus late windows), objective measures of compliance (e.g., wearing a camera), and long follow-up (<12 months). In addition, trials should report medication changes, baseline BMI and insulin use to allow subgroup analysis.

In conclusion, TRE may provide modest benefits in T2DM with respect to HbA1c and waist circumference; however, the evidence is limited by very high heterogeneity, uncertainty in pooled estimates, and low-to-moderate GRADE reliability. Subgroup analyses were insufficient to identify the sources of the differences, and sensitivity analyses showed that several important findings were highly dependent on individual studies. Therefore, these results are purely hypothesis-generating and do not support the final clinical recommendations. There is an urgent need for large-scale, long-term RCTs with standardized protocols.

## Data Availability

The datasets presented in this study can be found in online repositories. The names of the repository/repositories and accession number(s) can be found in the article/Supplementary material.
