# Supplementary material for: Effects of time-restricted eating on metabolic panel and sleep profile in patients with type 2 diabetes: a meta-analysis
Source: Front Nutr. 2026 Sep 14;13:1906991. doi: 10.3389/fnut.2026.1906991 (PMC13617046; doi:10.3389/fnut.2026.1906991)
Supplement: Supplementary file 1 [file Data_Sheet_1.PDF]

Supplementary Table 1:

full search strings for each database:

PubMed

#1 "Diabetes Mellitus, Type 2"[MeSH Terms]

OR "Diabetes Mellitus, Type 2"[Title/Abstract]

OR "T2DM"[Title/Abstract]

OR "Noninsulin Dependent Diabetes Mellitus"[Title/Abstract]

OR "Non-Insulin-Dependent Diabetes Mellitus"[Title/Abstract]

OR "Maturity-Onset Diabetes Mellitus"[Title/Abstract]

OR "Adult-Onset Diabetes Mellitus"[Title/Abstract]

OR "Ketosis-Resistant Diabetes Mellitus"[Title/Abstract]

OR "NIDDM"[Title/Abstract]

OR "Type 2 Diabetes"[Title/Abstract]

OR ("type 2"[Title/Abstract] AND (diabet*[Title/Abstract] OR "Diabetes Mellitus"[Title/Abstract]))

#2 "time restricted feeding"[Title/Abstract]

OR "time restricted eating"[Title/Abstract]

OR "time restricted diet"[Title/Abstract]

OR "time restricted fasting"[Title/Abstract]

OR "time restricted meal intake"[Title/Abstract]

OR "TRE"[Title/Abstract]

OR "TRF"[Title/Abstract]

OR ("time restricted"[Title/Abstract] AND (feed*[Title/Abstract] OR diet*[Title/Abstract] OR fast*[Title/Abstract] OR meal*[Title/Abstract]))

#3 #1 AND #2

Embase

#1 'non-insulin dependent diabetes mellitus'/exp

OR 'type 2 diabetes mellitus'/exp

#2 "type 2 diabetes":ti,ab,kw

OR "T2DM":ti,ab,kw

OR "NIDDM":ti,ab,kw

OR "non insulin dependent diabetes":ti,ab,kw

OR ("type 2" NEAR/3 diabet*):ti,ab,kw

#3 #1 OR #2

#4 'time restricted feeding'/exp

#5 "time restricted feeding":ti,ab,kw

OR "time restricted eating":ti,ab,kw

OR "time restricted diet":ti,ab,kw

OR "time restricted fasting":ti,ab,kw

OR "time restricted meal intake":ti,ab,kw

OR ("time restricted" NEAR/3 (feed* OR eat* OR diet* OR fast* OR meal*)):ti,ab

OR "TRF":ti,ab,kw

OR "TRE":ti,ab,kw

#6 #4 OR #5

#7 #3 AND #6

Web of Science

#1 TS = ("Diabetes Mellitus, Type 2" OR "T2DM" OR "Noninsulin Dependent Diabetes Mellitus"

OR "Non-Insulin-Dependent Diabetes Mellitus" OR "Maturity-Onset Diabetes Mellitus"

OR "Adult-Onset Diabetes Mellitus" OR "Ketosis-Resistant Diabetes Mellitus"

OR "NIDDM" OR "Type 2 Diabetes"

OR ("Type 2" NEAR/3 (Diabet* OR "Diabetes Mellitus")))

#2 TS = ("Time Restricted Feeding" OR "Time Restricted Eating" OR "Time Restricted Diet"

OR "Time Restricted Fasting" OR "Time Restricted Meal Intake"

OR "TRF" OR "TRE"

OR ("time restricted" NEAR/3 (feed* OR eat* OR diet* OR fast* OR meal*)))

#3 #1 AND #2

Cochrane Library

#1 [MeSH DESCRIPTOR] "Diabetes Mellitus, Type 2" EXPLODE ALL

#2 "diabetes mellitus, type 2":ti,ab,kw

OR "t2dm":ti,ab,kw

OR "noninsulin dependent diabetes mellitus":ti,ab,kw

OR "non-insulin-dependent diabetes mellitus":ti,ab,kw

OR "maturity-onset diabetes mellitus":ti,ab,kw

OR "adult-onset diabetes mellitus":ti,ab,kw

OR "ketosis-resistant diabetes mellitus":ti,ab,kw

OR "niddm":ti,ab,kw

OR "type 2 diabetes":ti,ab,kw

OR ("type 2" NEAR/3 diabet*):ti,ab,kw

#3 #1 OR #2

#4 "time restricted feeding":ti,ab,kw

OR "time restricted eating":ti,ab,kw

OR "time restricted diet":ti,ab,kw

OR "time restricted fasting":ti,ab,kw

OR "time restricted meal intake":ti,ab,kw

OR ("time restricted" NEAR/3 (feed* OR eat* OR diet* OR fast* OR meal*)):ti,ab,kw

OR "TRF":ti,ab,kw

OR "TRE":ti,ab,kw

#5 #3 AND #4

Scopus

#1 TITLE-ABS-KEY ("Diabetes Mellitus, Type 2" OR "T2DM"

OR "Noninsulin Dependent Diabetes Mellitus"

OR "Non-Insulin-Dependent Diabetes Mellitus"

OR "Maturity-Onset Diabetes Mellitus" OR "Adult-Onset Diabetes Mellitus"

OR "Ketosis-Resistant Diabetes Mellitus" OR "NIDDM"

OR "Type 2 Diabetes"

OR ("Type 2" W/3 (Diabet* OR "Diabetes Mellitus")))

#2 TITLE-ABS-KEY ("time restricted feeding" OR "time restricted eating"

OR "time restricted diet" OR "time restricted fasting"

OR "time restricted meal intake"

OR ("time restricted" W/3 (feed* OR eat* OR diet* OR fast* OR meal*)))

#3 #1 AND #2

Supplementary Figures.

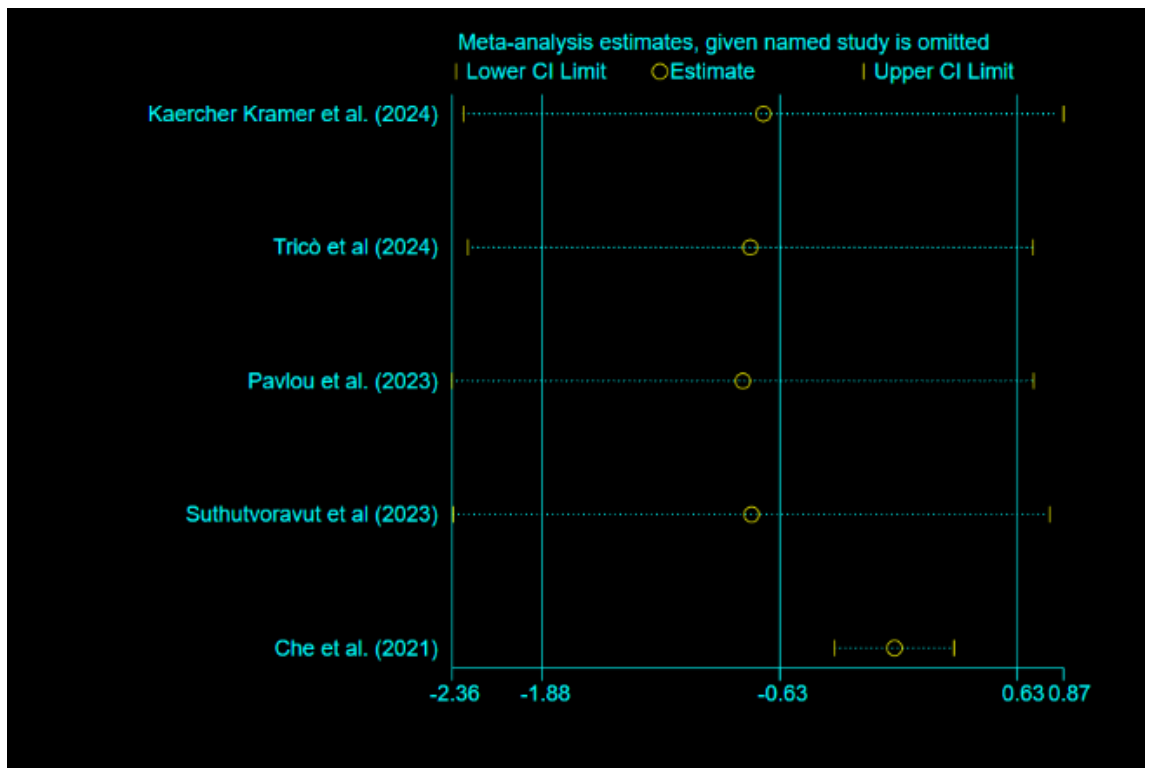

Supp. Figure 1.A. Sensitivity analysis for FBS.

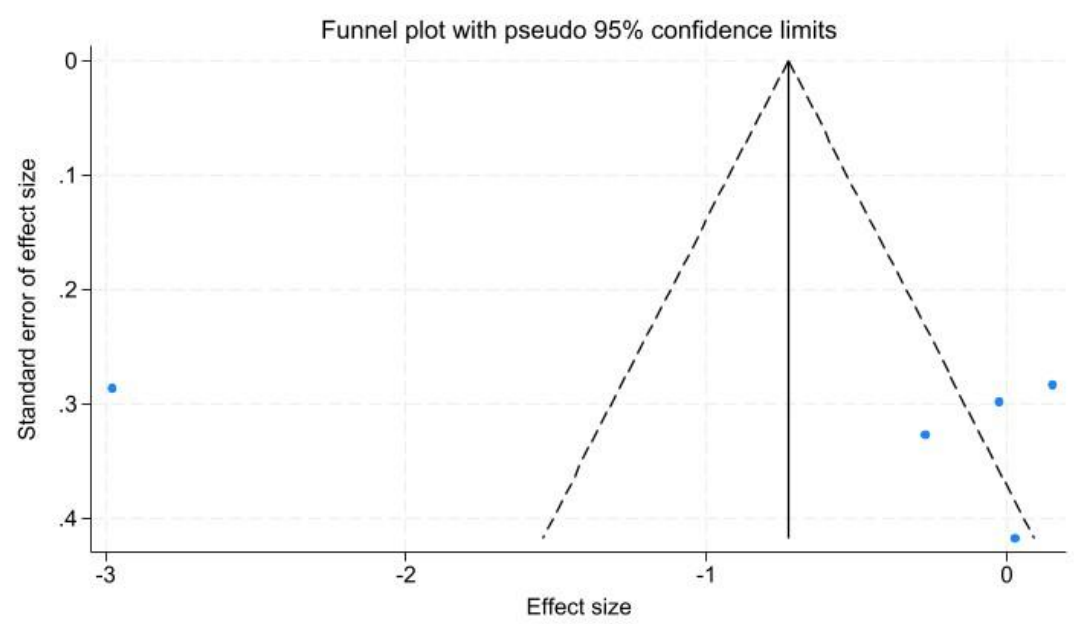

Supp. Figure 1.B. Publication bias for FBS.

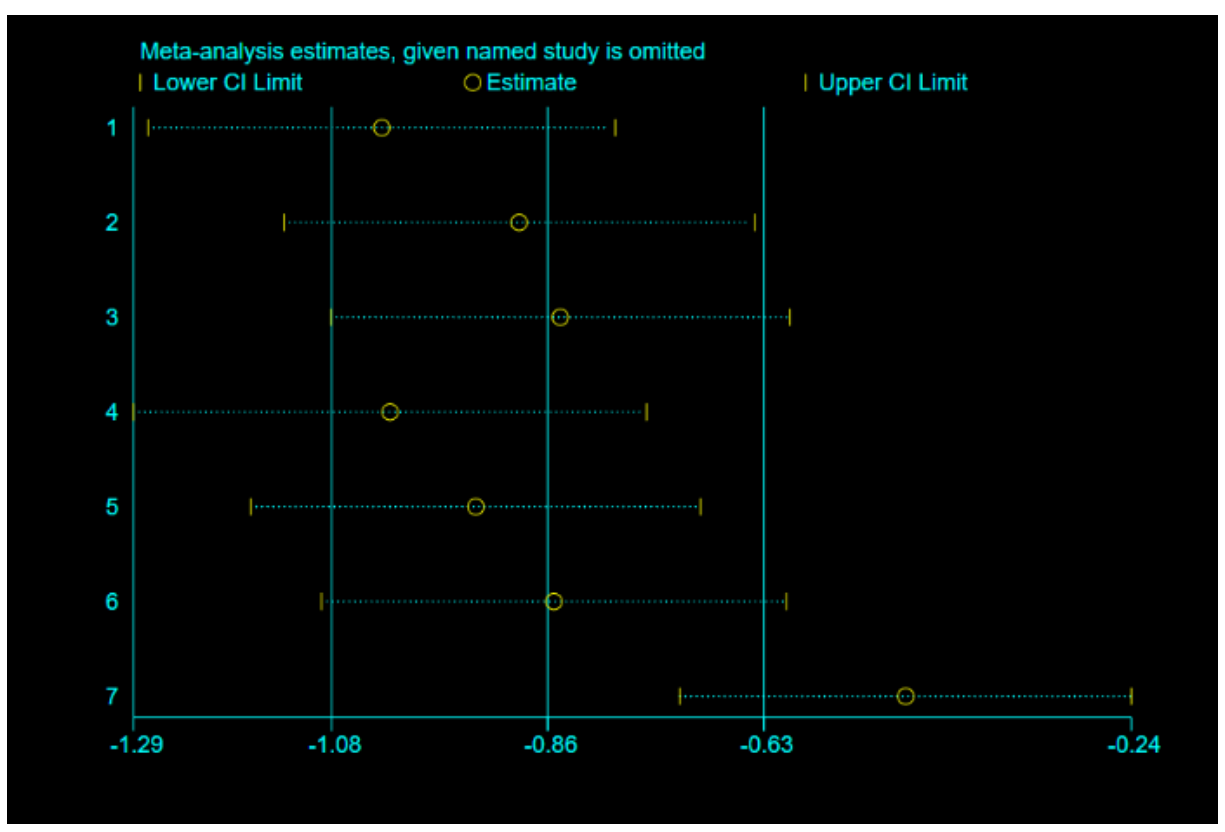

Supp. Figure 1.C. Sensitivity analysis for HbA1c.

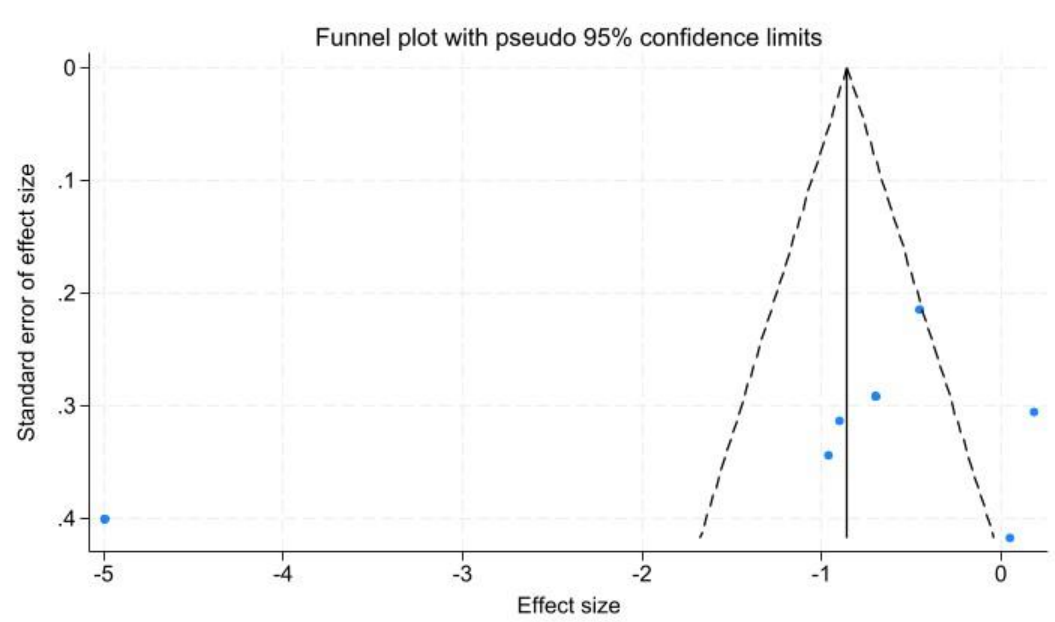

Supp. Figure 1.D. Publication bias for HbA1c.

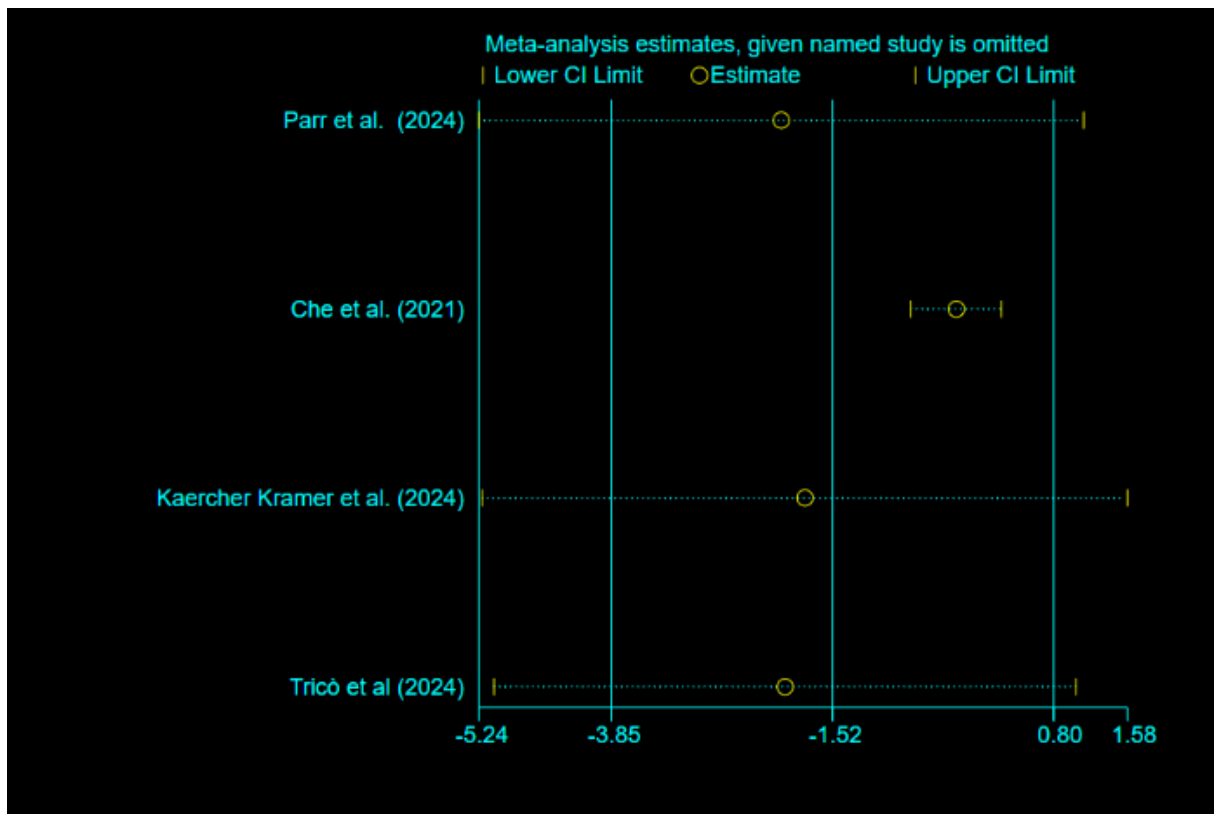

Supp. Figure 1.E. Sensitivity analysis for HOMA-IR.

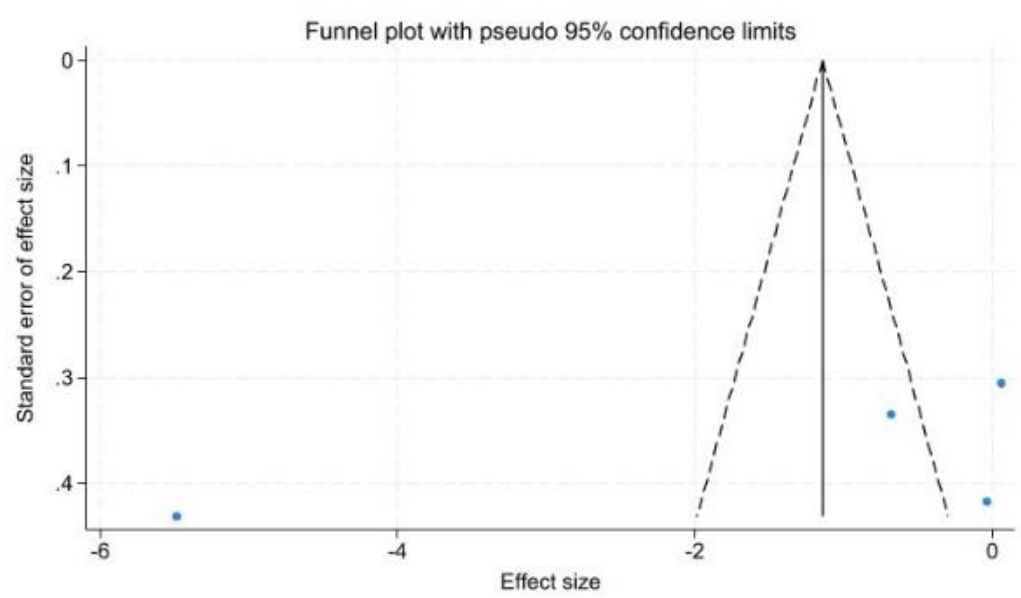

Supp. Figure 1.F. Publication bias for HOMA-IR.

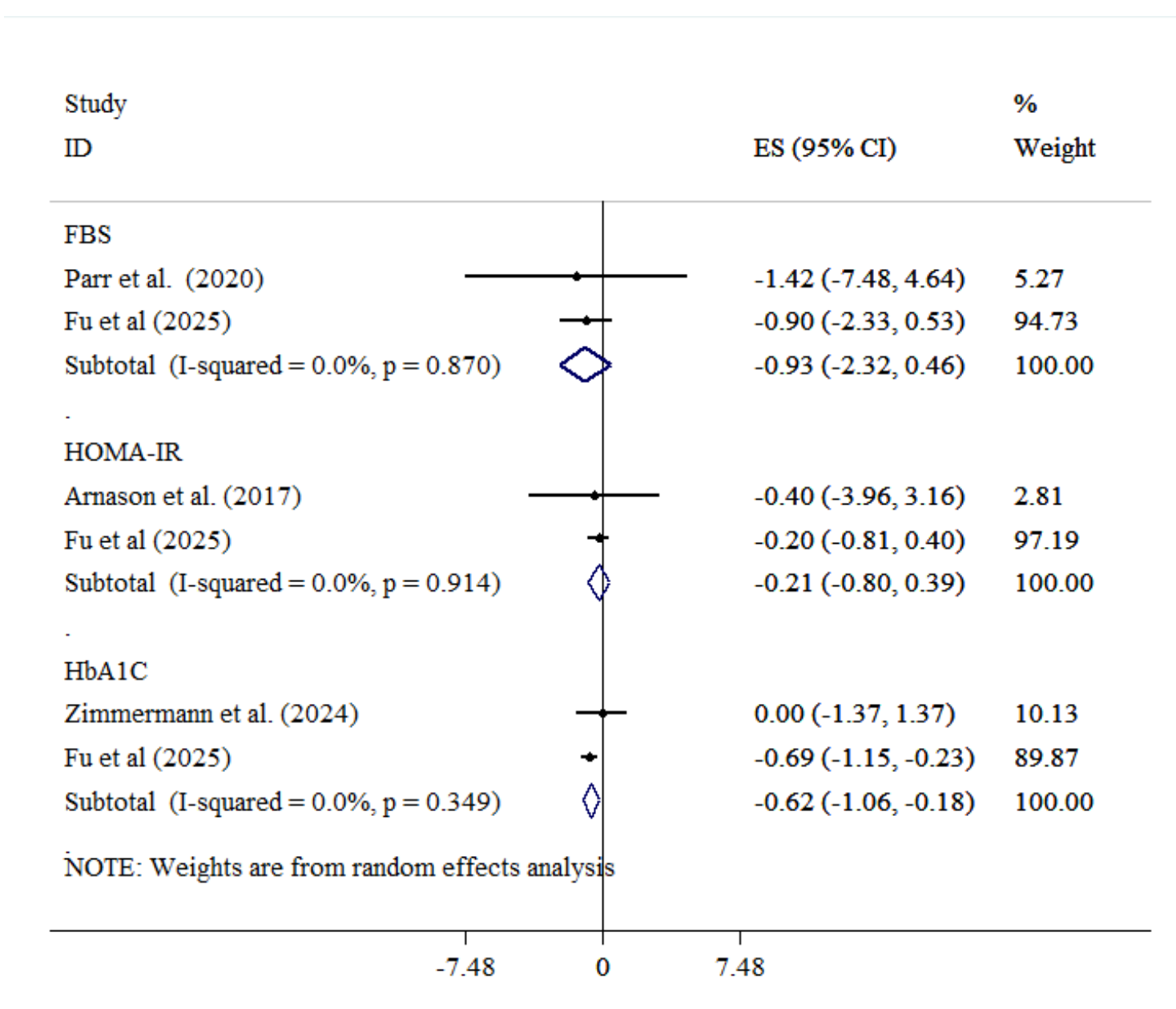

Supp. Figure 2. Forest plot of glycemic markers for BAS model.

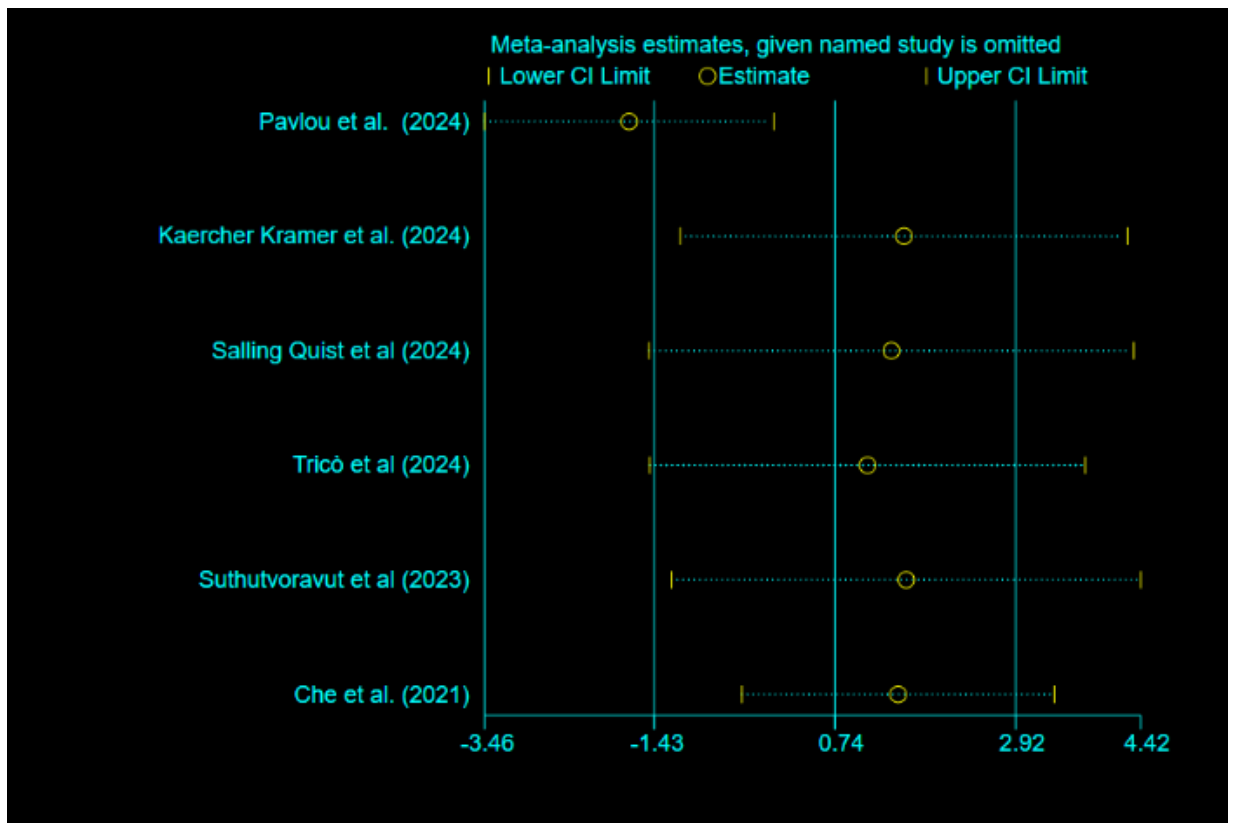

Supp. Figure 3.A. Sensitivity analysis for weight.

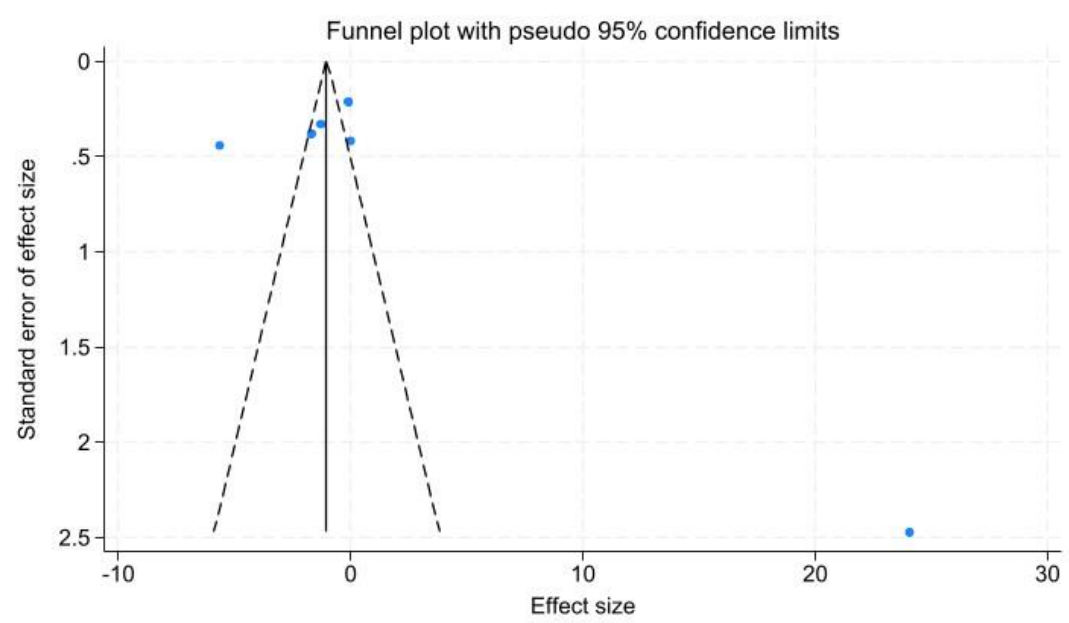

Supp. Figure 3.B. Publication bias for weight.

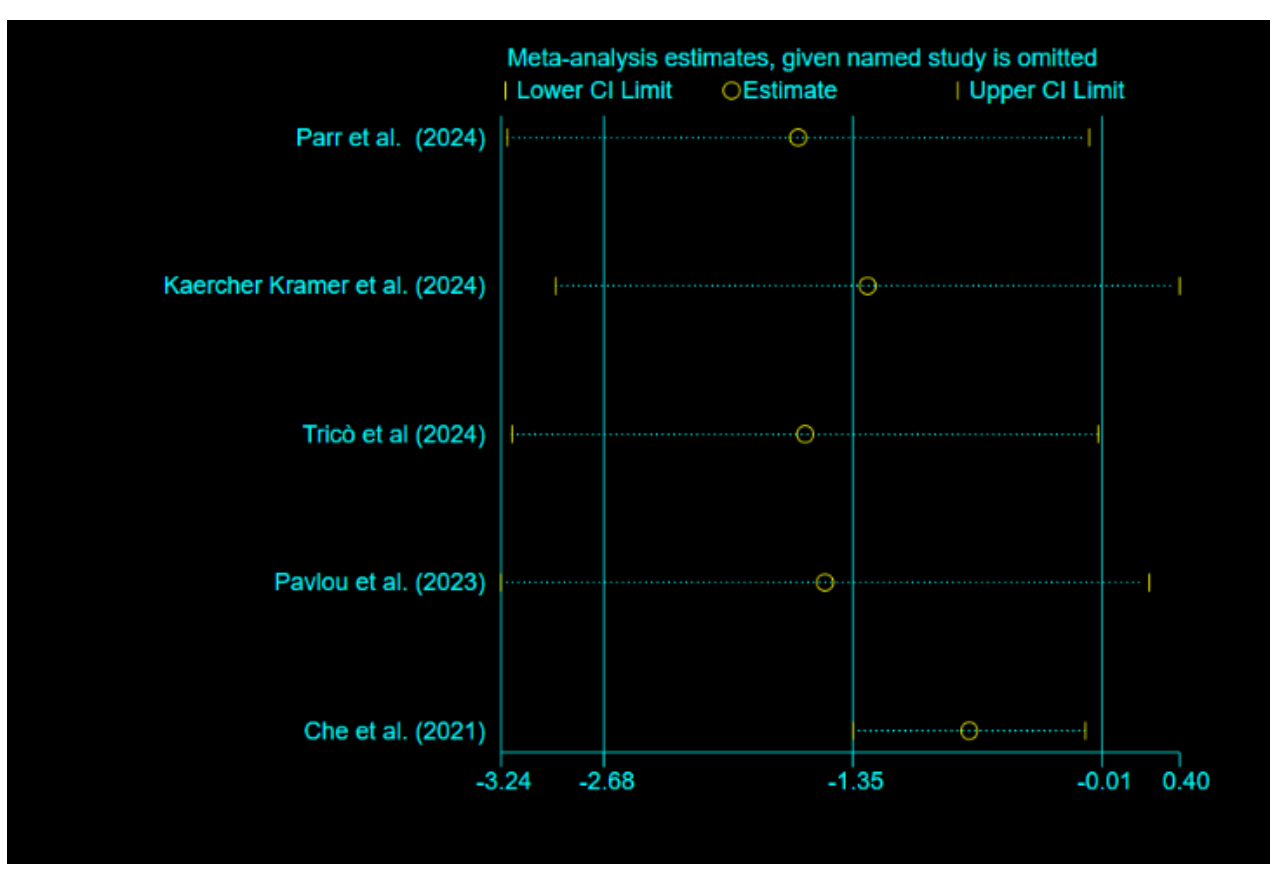

Supp. Figure 3.C. Sensitivity analysis for BMI.

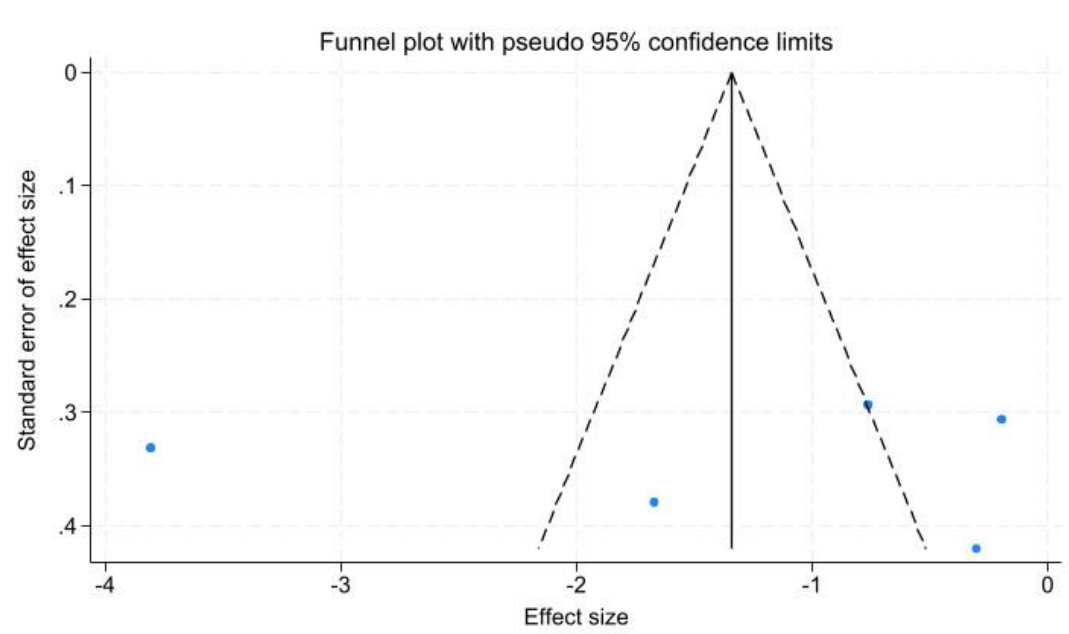

Supp. Figure 3.D. Publication bias for BMI.

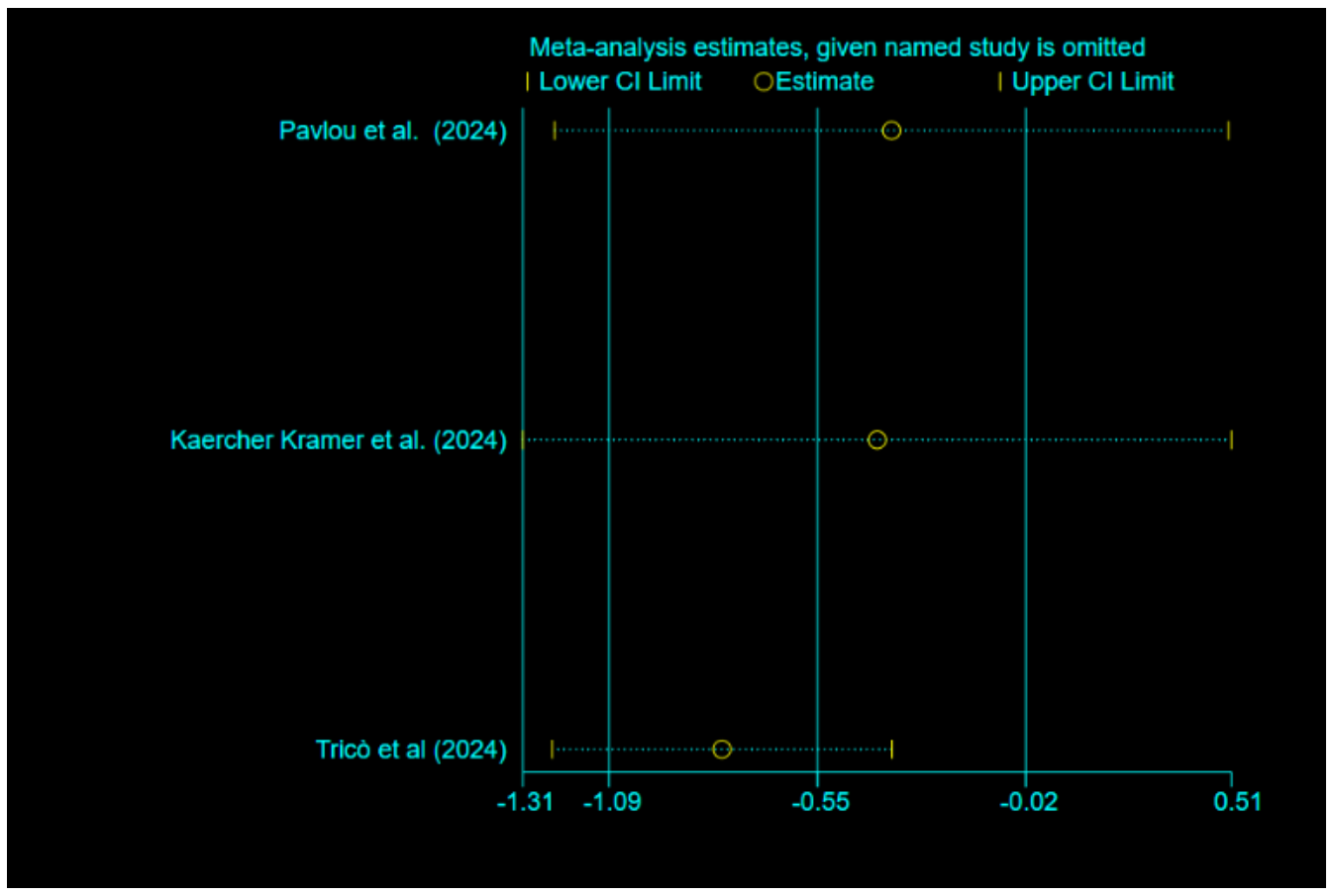

Supp. Figure 3.E. Sensitivity analysis for WC.

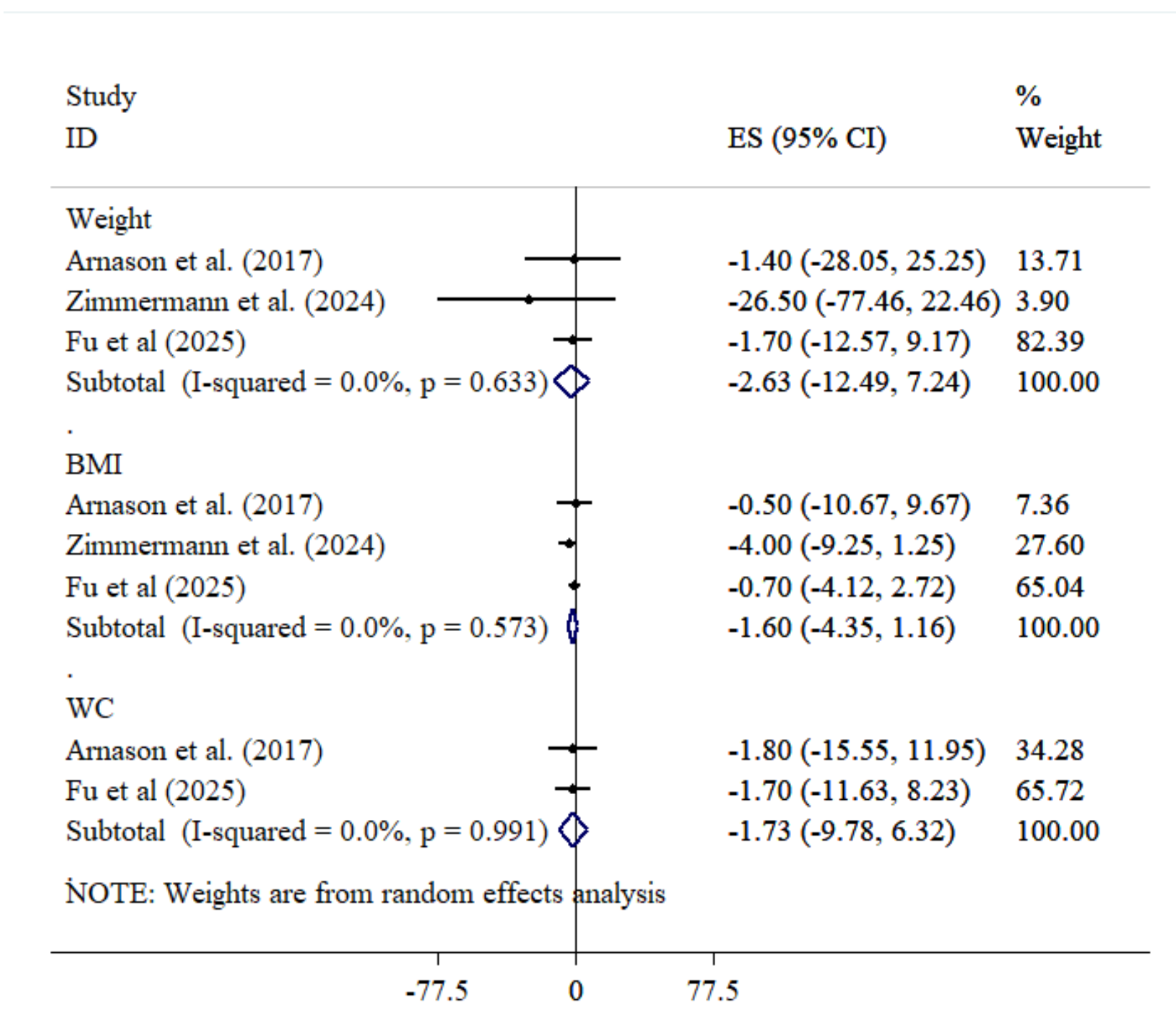

Supp. Figure 4. Forest plot of anthropometric indices for BAS model.

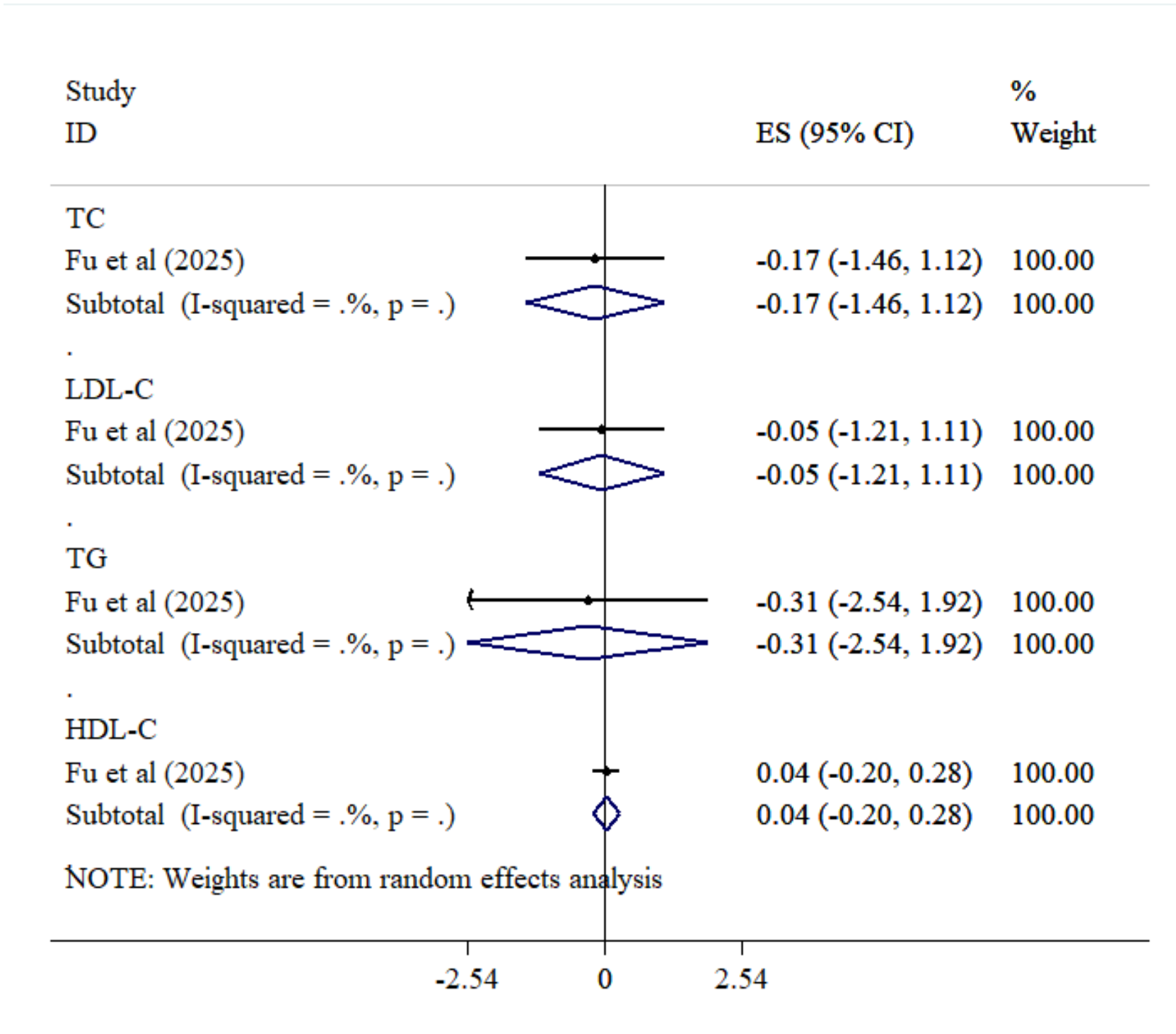

Supp. Figure 5. Forest plot of lipid profile for BAS model.

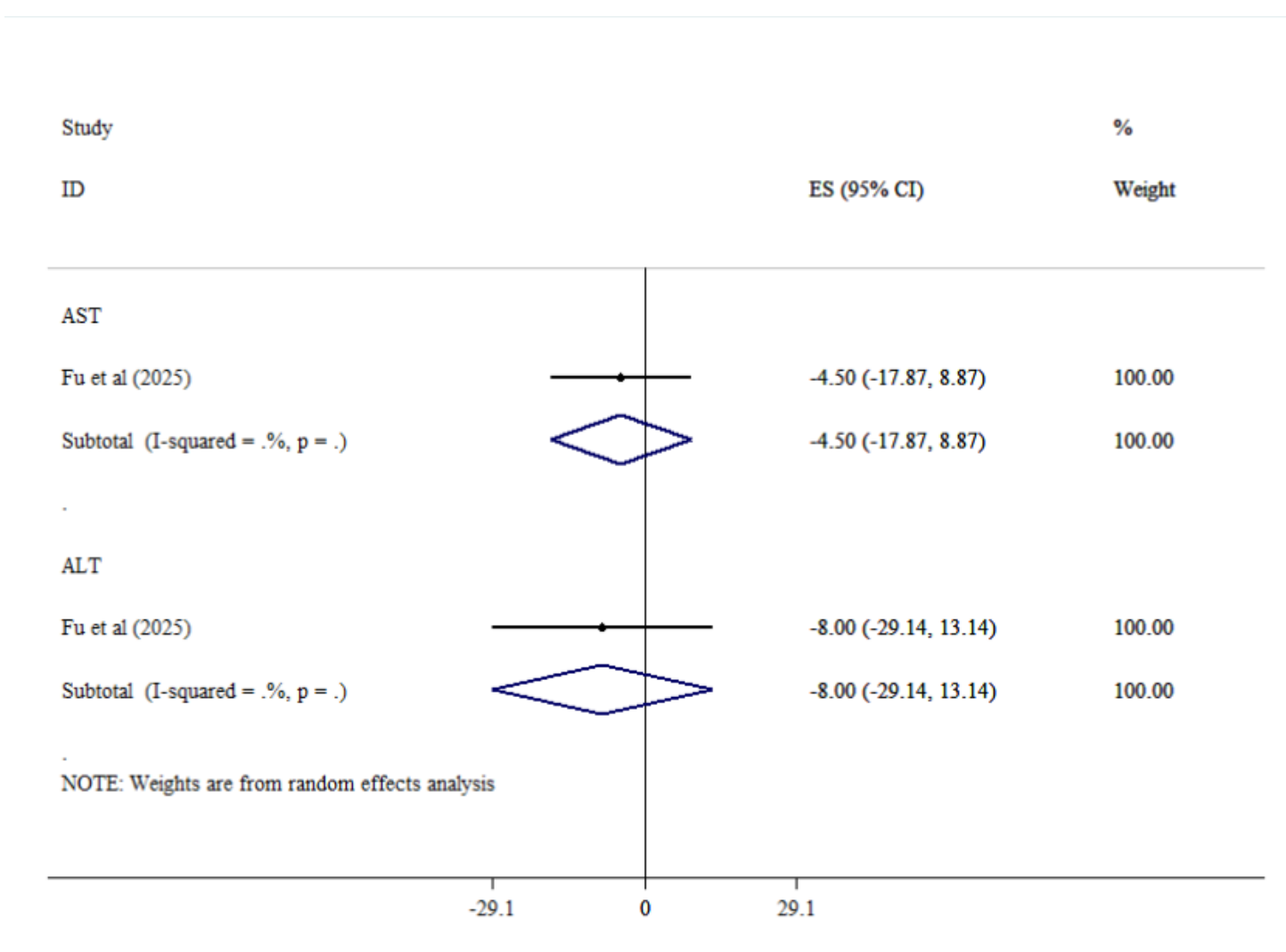

Supp. Figure 6. Forest plot of liver markers for BAS model.

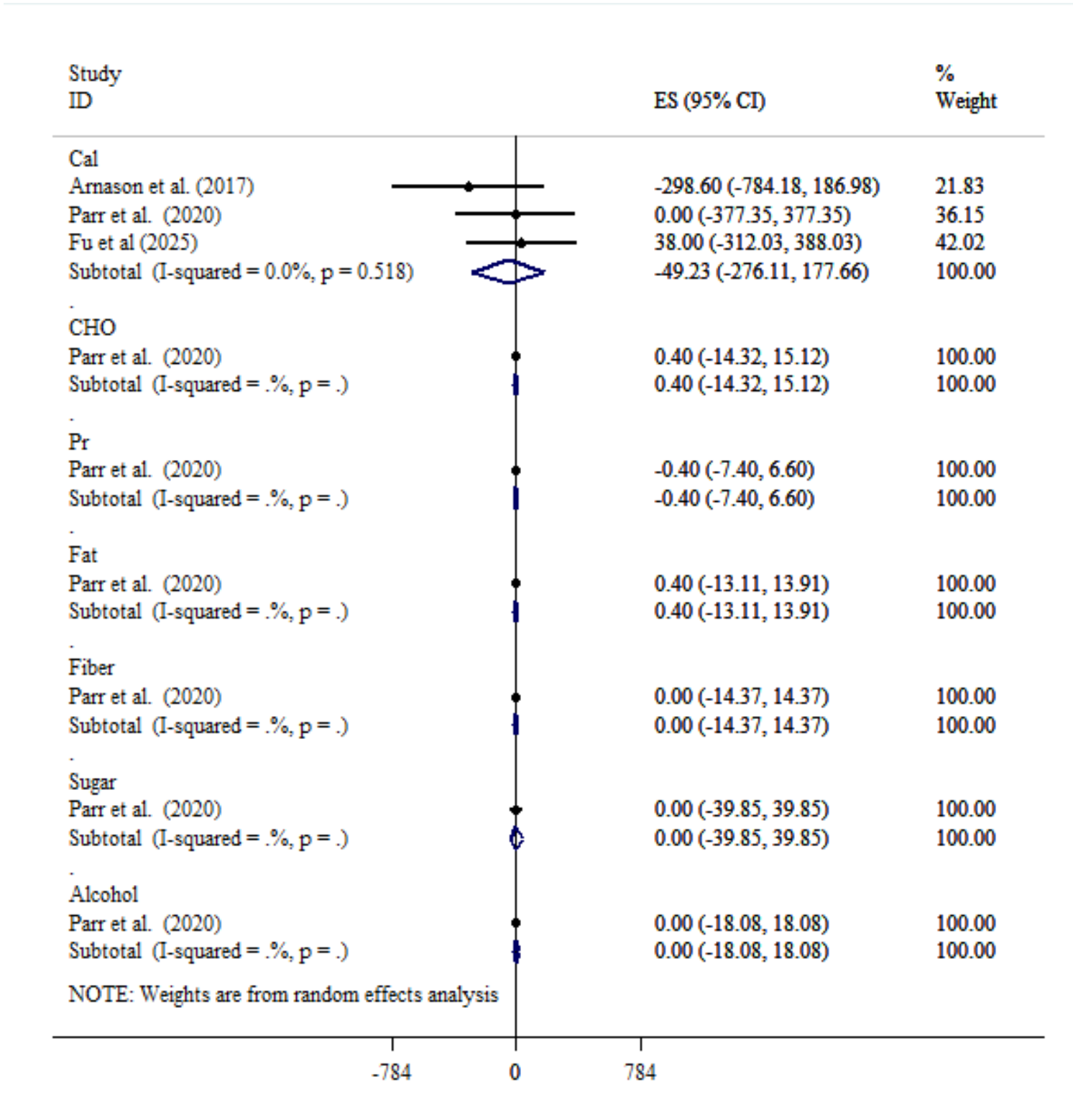

Supp. Figure 7. Forest plot of dietary intakes for BAS model.

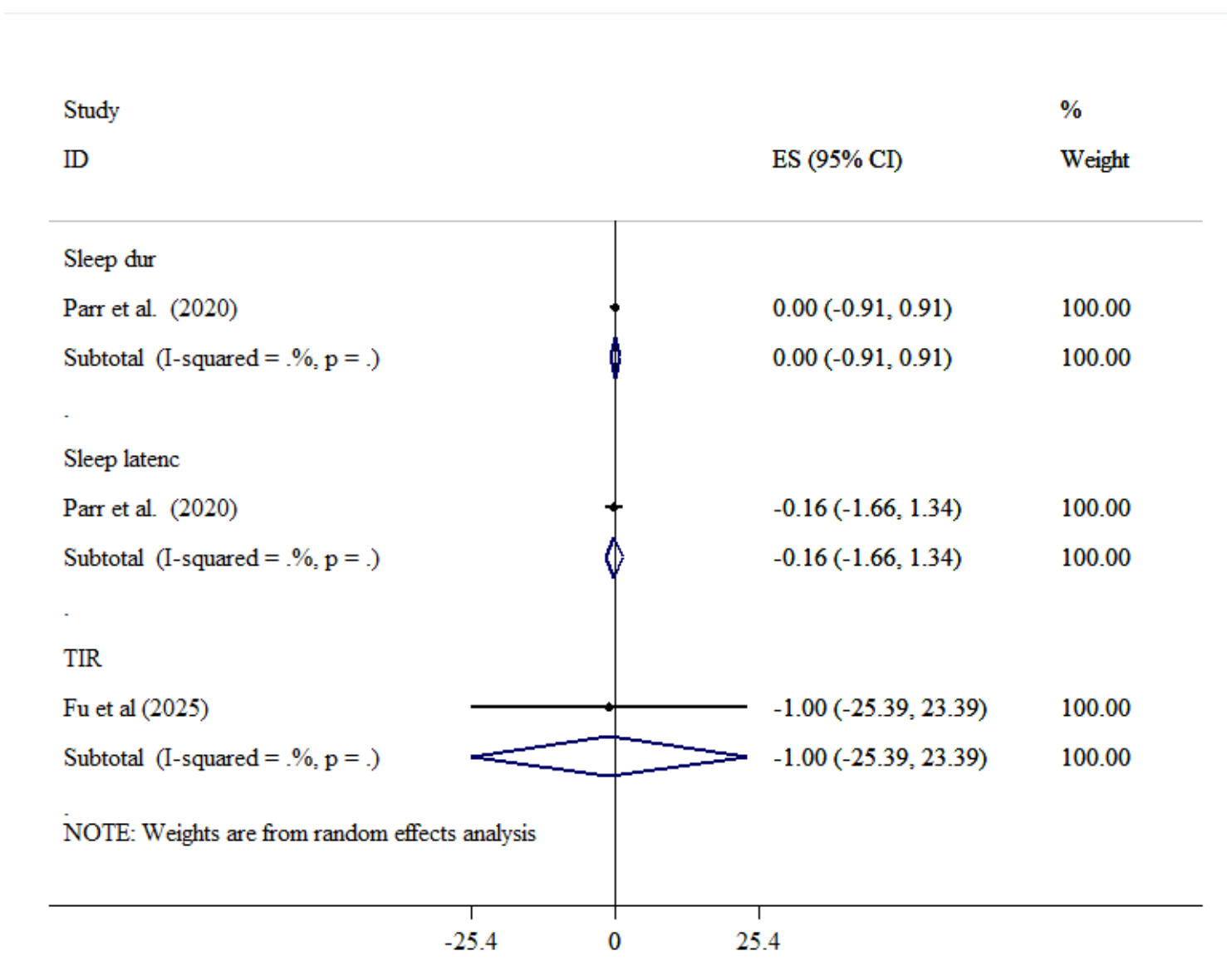

Supp. Figure 8. Forest plot of sleep outcomes for BAS model.
